# Mass vaccination and drug administration for malaria elimination in Bangladesh: design and implementation of a community engagement strategy

**DOI:** 10.1186/s40900-026-00995-1

**Published:** 2026-10-29

**Authors:** Md Ibrahim Khalil, Md Fojle Rabby, Dewan Imtiaj Ahmed, Bipin Adhikari, Thomas J. Peto, Phaik Yeong Cheah, Sazid Ibna Zaman, Richard J. Maude, Md Amir Hossain, A. K. M. Fazlur Rahman, Rasheda Samad, Aniruddha Ghose, Md Halimur Rashid, Md Mushfiqur Rahman, Rumana Rashid, Md Akramul Islam, Nicholas P. J. Day, Arjen M. Dondorp, Lorenz von Seidlein, Md Abul Faiz, Rupam Tripura

**Affiliations:** 1 Dev Care Foundation, Dhaka, Bangladesh; 2 Mahidol-Oxford Tropical Medicine Research Unit, Faculty of Tropical Medicine, Mahidol University, Bangkok, Thailand; 3 Centre for Tropical Medicine and Global Health, Nuffield Department of Clinical Medicine, University of Oxford, Oxford, UK; 4 The Open University, Milton Keynes, UK; 5 Centre for Injury Prevention and Research, Bangladesh (CIPRB), Dhaka, Bangladesh; 6 National Malaria Elimination Program, Ministry of Health and Family Welfare, Dhaka, Bangladesh; 7 Bangladesh Rural Advancement Committee, Dhaka, Bangladesh

**Keywords:** Community engagement, Malaria, Mass drug administration, Vaccination, South Asia

## Abstract

**Background:**

Community engagement is increasingly recognised as an essential element of health research in underserved settings, yet limited evidence describes how engagement strategies are designed, adapted, and implemented during large-scale intervention trials. This implementation case study describes the development, implementation, and iterative adaptation of a stakeholder and community engagement strategy embedded within a field trial of mass vaccination and drug administration (MVDA) for malaria elimination in Bangladesh.

**Methods:**

The cluster-randomised, open-label trial was conducted across 100 villages (~12,000 people) in the Chittagong Hill Tracts, Bangladesh. The paper draws on implementation records, field observations, meeting reports, and structured team reflections generated during programme delivery to describe the engagement process, key adaptations, implementation challenges, and lessons learned.

**Results:**

Early stakeholder engagement informed a context-specific engagement strategy that emphasised collaboration with village leaders, culturally tailored communication, responsiveness to community concerns, and integration of basic healthcare support. Throughout implementation, engagement activities were iteratively adapted in response to community feedback, rumours, seasonal livelihood demands, and local cultural and religious beliefs. These iterative approaches were followed by continued engagement across remote and culturally diverse communities, although persistent challenges included geographical isolation, social and political factors, complex trial messaging, competing livelihood priorities, and concerns regarding vaccination, including fears about fertility.

**Conclusions:**

Community engagement functioned as an adaptive and responsive process rather than a fixed intervention. These experiences provide transferable lessons for designing stakeholder and community involvement strategies for complex health research conducted in underserved and culturally diverse settings.

**Supplementary information:**

The online version contains supplementary material available at https://doi.org/10.1186/s40900-026-00995-1.

## Abstract (fr)

Community engagement is an important part of health research, especially when studies involve whole communities. It helps researchers understand local concerns, build trust, and work together with communities to improve how research is carried out. However, there is limited information about how community engagement is designed and adapted during large research projects in remote and culturally diverse settings. This paper describes how community engagement was developed and implemented during the first field trial of mass malaria vaccination and drug administration in Bangladesh. The study took place in 100 villages in the Chittagong Hill Tracts, where many people belong to different ethnic groups, speak different languages, and have limited access to healthcare. Before the study began, we worked with government officials, health workers, village leaders, religious leaders, and community members to understand local priorities and concerns. Their feedback helped us develop culturally appropriate information materials and engagement activities. Throughout the study, we continued to listen to community members and adapted our approach when new concerns arose. For example, we responded to rumours about infertility and vaccine safety, adjusted activities around farming and religious festivals, worked closely with trusted local leaders, and provided basic healthcare where possible. These approaches were followed by continued engagement and, in some cases, subsequent participation, although challenges remained because of remote geography, social and political history of the region, seasonal livelihood pressures, and cultural beliefs. Our experience shows that community engagement should not be viewed as a one-time activity. Instead, it should be an ongoing, flexible process that responds to community needs and changing circumstances. These lessons may help researchers design more effective and respectful community engagement for future health research in underserved communities.

## Introduction

Substantial progress has been made toward malaria elimination in Asia. Community engagement remains an important component of ethical global health research in underserved communities [1]. The R21/Matrix-M vaccine prevents Plasmodium falciparum malaria infection by stimulating production of antibodies against the sporozoite stage of the parasite [2]. WHO recommended R21/Matrix-M in October 2023 for the prevention of malaria in children living in malaria-endemic areas and prequalified the vaccine in December 2023 [3]. The present trial evaluates the vaccine in a broader population than this pediatric recommendation. However, many countries have not yet achieved interruption of transmission, particularly among remote, rural populations at risk [4].

Population-level interventions aiming to accelerate malaria elimination have been implemented in many settings, most commonly mass drug administration (MDA), in which entire communities take antimalarial drugs to reduce the asymptomatic parasite reservoir [5]. MDA can be effective in diverse settings, but high population coverage is generally needed to reduce the risk of resurgence [6]. Even with good coverage, population movements can reintroduce malaria transmission as the drugs used have a curative effect but do not provide prolonged protection in the absence of continuous prophylaxis. For MDA and similar population-level interventions to be ethical, feasible, and acceptable, they must be implemented in ways that are responsive to local social and cultural contexts [7, 8]. Community engagement is therefore critical for understanding community priorities, addressing concerns, and adapting implementation to local contexts [9–11]. Despite increasing recognition of its importance, relatively few studies describe how engagement strategies evolve during complex intervention trials, particularly in geographically remote and culturally diverse and politically sensitive settings.

In February 2025, the first field trial of R21 in Bangladesh began, providing an opportunity to examine stakeholder and community engagement within a complex malaria elimination intervention. The cluster-randomised, open-label Mass Vaccination and Mass Drug Administration (MVDA) trial was implemented across 100 small villages in the Chittagong Hill Tracts (CHT), close to the border with Myanmar and in the main remaining region of Bangladesh where malaria remains a significant health burden. Preparatory early discussions (stakeholder engagement) began before the intervention, with consultations and planning activities continuing through 2024.

The preparatory work included formative qualitative research in March–May 2023 and a separate baseline qualitative/KAP study in July–October 2024 [12]. The detailed qualitative findings are reported separately. Briefly, these studies highlighted the linguistic and cultural diversity of the CHT, seasonal jhum (shifting) cultivation, limited access to formal healthcare, the importance of local leadership and community structures for engagement, and historical mistrust of outsiders [13]; these contextual features are described further below.

The combination of remoteness, linguistic and cultural diversity, political history, and reliance on community-based structures required an adaptive engagement process that protected participants’ interests and responded to community priorities [1, 14]. Although community engagement has been widely discussed, little is known about how engagement at this scale is designed and adapted in remote, politically isolated, and ethnically diverse communities. The main objective of this study was to describe the development, implementation, and iterative adaptation of stakeholder and community engagement within the MVDA trial and synthesize practical lessons for future community-engaged research in similar settings.

## Materials and methods

### Description of the MDVA study

**Fig. 1:**
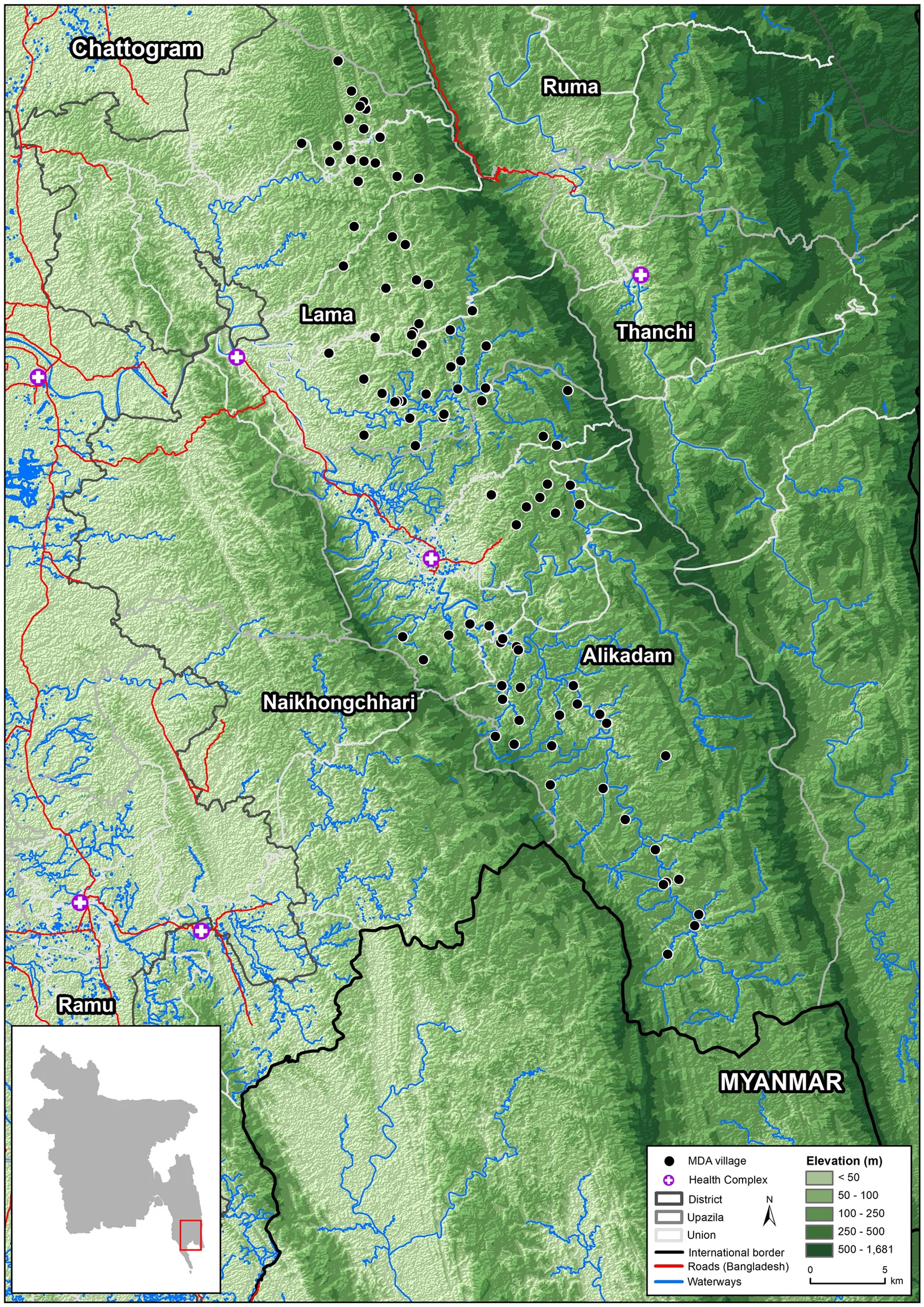
Study sites map visualizing study villages within CHT district

This article presents an implementation case study describing the design, implementation, and adaptation of stakeholder and community engagement embedded within a cluster-randomised, open-label trial conducted in 100 villages with an estimated population of 12,000 in the CHT, Bangladesh (Fig. 1).

The MVDA trial is designed to evaluate the effectiveness of the intervention (ClinicalTrials.gov registration# NCT06068530, registered on 5th October 2023). The trial has a factorial design comparing four intervention arms: MVDA, Mass Vaccine Administration (MVA) alone, Mass Drug Administration (MDA) alone, and the current standard of care (control), which includes long-lasting insecticidal nets and early diagnosis and treatment. Interventions consist of three rounds of vaccination and/or MDA (three doses), with a 12-month booster in the vaccine arms, while control villages receive MVDA after two years. The trial results will be reported elsewhere.

### Social and cultural context of the study sites

Engagement activities were documented between 2023 and 2026 in villages inhabited primarily by ethnic minority communities including Mro, Tripura, Marma, and Tanchangya, alongside Bengali populations. Many residents lived in geographically isolated locations and moved between settlements for livelihood activities. The Chittagong Hill Tracts (CHT), comprising Rangamati, Khagrachari, and Bandarban, are characterised by marked ecological, linguistic, and cultural diversity and are home to around twelve indigenous communities [15, 16]. Traditional governance structures, particularly the Headman and Karbari (কারবারি) systems, continue to influence local leadership and community decision-making.

The contemporary social landscape of the CHT has been shaped by a complex history of political conflict, population displacement, state-sponsored settlement programmes, militarisation, and longstanding disputes over land ownership and resource access [13, 17]. These processes have contributed to experiences of marginalisation among indigenous communities and have influenced their relationships with government institutions and external actors. Traditional shifting cultivation (*jhum*), which historically formed the basis of many indigenous livelihoods, has increasingly been replaced or constrained by commercial agricultural activities, including tobacco cultivation and other cash crops [18]. At the same time, unresolved land claims, military presence, periodic episodes of violence, and reports of abduction involving ethnic minority populations continue to generate social and political grievances within the region [19].

The region’s contemporary social landscape has also been shaped by political conflict, population displacement, settlement programmes, militarisation, and longstanding disputes over land and resources. These historical and structural conditions have contributed to marginalisation and have influenced relationships with government institutions and external actors. These features had to be accounted and accommodated in designing and adapting engagement activities throughout the study [20].

### Research team organization, roles, training and reflexivity

Because of the scale and breadth of the villages included in the study, the Principal Investigator (PI) and coordinator established a research team during the preparatory stage [21]. Building on previous community-based mass antimalarial studies conducted in Cambodia [22], Laos [23], Vietnam [24], and the Thai–Myanmar border [25], the site coordinator recruited staff with defined roles and local familiarity relevant to operationalizing the MVDA study. The team included engagement staff, health workers, anthropological experts, and implementation researchers. Engagement with community leaders, village heads, health workers, government stakeholders, and other local actors was organised through existing community and health-system structures, with the aim of incorporating stakeholder feedback into study implementation and making the approach socially and culturally feasible [26, 27].

In each village, the team worked with one Karbari (কারবারি), one CHW (MVDA staff), and one SS (BRAC staff), supervised by 31 engagement field assistants and 5 engagement group leaders. In total, the local team comprised 336 people who are directly involved in interacting with villagers. The multidisciplinary composition of the team was intentional, allowing community engagement activities to integrate local knowledge, clinical expertise, anthropology, and implementation experience.

This manuscript presents a practice-based implementation case study rather than a formal qualitative study. It draws on multiple sources generated during routine implementation between 2023 and 2026, including observation-based field notes, implementation reports, meeting records, and structured team reflections. These materials were produced during routine programme delivery and monitoring rather than through a study designed for primary qualitative data collection. Records were reviewed for material relevant to engagement planning, community concerns, implementation challenges, adaptations, and observed responses.

To develop this manuscript, core members of the implementation team (IK, FR, and RT) convened with investigators experienced in community engagement and implementation research (TJP, BA, RT and PYC) in a structured five-day reflective workshop in Kathmandu, Nepal. The team reviewed the different record types together to reconstruct the sequence of implementation, identify recurring concerns and adaptations, and synthesise lessons across sources. Triangulation involved comparing field observations, implementation reports, meeting records, and team reflections; differing interpretations were discussed among field staff and engagement researchers and refined through repeated discussion. In contrast to formal qualitative data analysis, the central aim was to synthesise practice-based knowledge about implementation processes, contextual adaptations, and lessons learned [28–30]. Because the implementation team was also involved in documenting and interpreting the experience, reflexivity was addressed through involvement of investigators with different roles and experience, comparison of interpretations against contemporaneous records, and explicit discussion of discrepant accounts. The eight case examples were purposively selected to illustrate variation in the challenges encountered and the range of adaptations used; they are illustrative rather than representative of all programme experiences.

### Analytical approach

#### Design, implementation and adaptation of engagement strategies

**Fig. 2:**
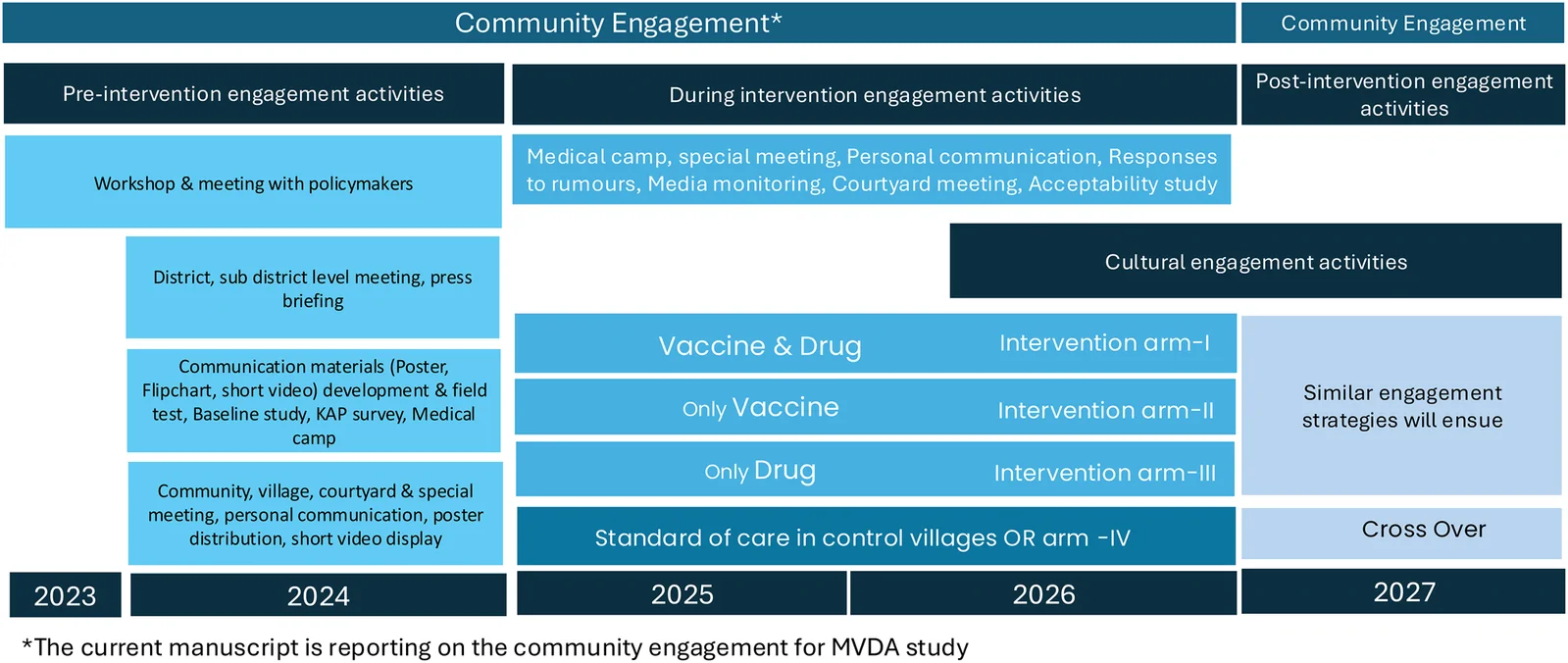
Schematic diagram of MVDA study and embedded community engagement

Stakeholder and community engagement was a core feature of the MVDA study (Fig. 2). The engagement included both activities aimed at enabling implementation and opportunities for stakeholders and community members to influence aspects of study implementation, communication materials, timing, local entry, and responses to emerging concerns. These activities cannot be equated with all forms of formal patient and public involvement (PPI) because of contextual differences. We therefore use the GRIPP2 framework to report relevant involvement activities while distinguishing community engagement, research participation, and formal PPI (Supplementary File 1) [31].

The overall community and stakeholder engagement strategy began with high-level workshops in Dhaka in 2023, involving medical experts, anthropologists, and communication consultants, as well as national-level policymakers from the National Malaria Elimination Programme, under Directorate General of Health Services (DGHS), Directorate General of Drug Administration (DGDA), Bangladesh Rural Advancement Committee (BRAC), Centre for Injury Prevention and Research, Bangladesh (CIPRB), Sisimpur, and other key organizations. A total of four workshops and meetings involving 65 participants were conducted from 2023 to 2024 (Supplementary File 2). These workshops also helped pre-test and finalize the engagement materials.

Following approval of the MVDA study, and feedback from these early and top-tier policymakers and stakeholders, the team engaged district- and sub-district-level authorities, including government officials, military personnel, police officers, journalists, and media representatives. This phase also involved proactive engagement with media and journalists to inform them about the study and prevent misinformed reporting.

Following these meetings, the team engaged union-level stakeholders, including village leaders, headmen, and religious leaders. Stakeholders were selected according to their administrative, health-service, community leadership, religious, media, or logistical roles in the study setting. During these discussions, the study was introduced and feedback was sought on study procedures, communication, timing, local concerns, and practical implementation. Perspectives from community members, leaders, health workers, and researchers were brought together during field discussions and the reflective synthesis, with differing interpretations discussed and reconciled where possible. Rather than evaluating the effectiveness of community engagement or applying a theory-of-change or input-process-output framework, this case study documents how engagement strategies were developed, adapted, and implemented throughout the research programme, with iterative adaptation as the organising feature. This aligned with a broad aim of generating a descriptive account of ground experience of implementing large scale community-based trial and the practicalities of community engagement.

#### Community engagement procedures, materials and activities

Community engagement was implemented through multiple complementary approaches rather than a single activity. Approaches were refined iteratively throughout implementation in response to stakeholder feedback and emerging challenges. Key communication materials included flipcharts, posters, videos, booklets, and frequently asked questions developed from questions raised during meetings and informal interactions (Supplementary File 3). Materials were developed and reviewed by the engagement and communication team with relevant technical and anthropological expertise, translated into Bengali and Mro, and supported by interpreters from the relevant communities for other languages. They were field-tested and refined through several sessions with community members to improve clarity, comprehensibility, and cultural appropriateness before wider dissemination.

At the community level, multiple engagement strategies were implemented to facilitate informed engagement across the study villages. Village meetings were conducted with *karbaris*, religious leaders, traditional healers, teachers, educated youths and adult community members across all 100 villages. The team also displayed short videos 170 times in 100 villages, covering 3,837 adults, using a portable projector or mobile phone. Simultaneously, 2,218 large posters were pasted on the walls of households, and 4,610 A4-sized posters were distributed among the villagers. These were complemented by 735 courtyard meetings with 6723 individuals. Each courtyard meeting involved 7–15 adults, was conducted in a village and was led by local health workers and engagement field assistants. Additionally, house-to-house visits reached approximately 4,000 individuals. Together, these approaches provided multiple opportunities for direct communication, clarification of concerns, and informed decision-making. The activity counts describe the scale of engagement and may include repeated contacts across activities; they are not meant to be interpreted as counts of unique individuals or as population coverage.

In addition to formal activities, informal approaches were employed, such as engaging with potential participants at market gatherings, cultural events (e.g. weddings, cow-killing ceremonies, and funerals), and through one-to-one interactions. Although these were outside the formal engagement strategy, they provided additional opportunities for dialogue, relationship-building, and identification of emerging concerns. They also allowed the team to respond to rumours and implementation problems as they arose.

Additional engagement strategies (referred to as special meetings within the study team) were implemented in 20 villages where emerging concerns, rumours, or other circumstances indicated a need for additional dialogue. These meetings involved local leaders and other relevant actors to explore concerns, clarify study information, and gather feedback.

Based on community suggestions, discussions with village leaders, and field observations of substantial unmet health needs, basic healthcare was incorporated into the community-based study approach in all study villages. These services were delivered by MVDA health workers (dedicated health workers recruited for MVDA intervention) in collaboration with government healthcare facilities and workers. They covered conditions such as scabies, diarrhoea, the common cold, back and joint pain, cuts and accidental injuries, burns, snakebites, dog bites, and other illnesses.

At one point, a scabies outbreak occurred in almost all villages, and the study team provided treatment at scale, after which the team reported containment of scabies transmission. Similarly, diarrhoea was a life-threatening condition in the villages and had claimed approximately 5–7 lives in previous years. In response, the team proactively provided oral rehydration solution for immediate treatment. These activities also formed part of the practical context in which engagement took place.

The team collaborated with the Expanded Programme on Immunization (EPI) to align vaccination activities with the MVDA study procedures and avoid interference. This collaboration also facilitated continuous feedback between communities and service providers.

The study also included regular media monitoring to identify and mitigate rumours, alongside frequent feedback meetings with field teams to enable proactive responses.

### Ethical considerations

This study is part of the Mass Vaccine and Drug Administration (MVDA) study in Bangladesh and adheres to its established protocol. The research was conducted in accordance with the ethical principles of the Declaration of Helsinki. Ethical approval was obtained from the Bangladesh Medical Research Council (Reference: BMRC/NREC/2022-2025/268) and the Oxford Tropical Research Ethics Committee (OxTREC: 37-23); Clinical Trial Registration Number: NCT06068530. This study reports stakeholder and community engagement activities conducted during both the preparatory phase and the intervention period. Community-level permission or authorization supported entry, dialogue, and engagement but did not substitute for individual informed consent. Community leaders, including karbaris, could not consent on behalf of individual participants. Where study-related data were collected, standard written informed consent was obtained before participation. Participation was voluntary, and individuals could decline participation without affecting access to health services or other study-related care. Engagement activities and any support offered to reduce practical access barriers were intended to facilitate informed decision-making rather than compel participation.

## Results

### Stakeholder recommendations and initial adaptations

#### Tailor communication tools for communities

For development of communication materials, stakeholders recommended using posters and videos as visual sources of information, tailored to local languages (Mro, Tripura and Bengali) and designed to be easily understandable for all. Some specific suggestions included incorporating TikTok as a popular social media platform although social media and internet are not accessible to some of the villages. Based on the piloting among non-MVDA village residents (*n* = 50), they recommended enlarging images in flipcharts and ensuring that visuals, such as pictures of medicines, are clearly recognizable and not confused with everyday items like soap, and the need for high-resolution images of rapid diagnostic test kits. The different non-MVDA village participants (*n* = 62) also suggested adding images of mosquitoes to reinforce the connection with malaria. This feedback was documented (Supplementary File 4) in addition to field notes and strategies from these meetings (Supplementary File 5). Participants also emphasised the importance of acknowledging traditional rituals and local knowledge and of communicating study information respectfully (Table 1).

**Table 1:** Early stakeholder input, engagement activities, and corresponding adaptations

Phase	Engagement input/activity	Who was engaged	Purpose/focus	Key adaptation or response
Pre-intervention	National stakeholder consultation	National policymakers, regulators, programme and NGO partners	Align study with policy and local context	Engage early; work through local leaders and health workers; plan around livelihoods; use local language and terminology; coordinate remote access.
Pre-intervention	Communication material development and field testing (112 adults from non-MVDA villages)	Communication, anthropology and technical experts; community members	Assess clarity, comprehensibility and cultural appropriateness	Enlarge and simplify visuals; add recognisable malaria and medicine images; translate/adapt materials for local languages.
Pre-intervention	District, sub-district and union orientation/media engagement	Government, health, elected, security and media stakeholders	Introduce study and clarify procedures, safety and operational roles	Promote consistent messages; clarify safety monitoring; schedule activities around festivals and cultivation; coordinate access without implying security ownership.
Pre-intervention	Formative and baseline qualitative/KAP work	Community members and stakeholders across study villages	Understand beliefs, perceptions, knowledge and barriers	Use findings to tailor language, trusted messengers, meeting formats and explanations of MVDA.
Pre-intervention	Village meetings (100 meetings; 4,095 adults recorded)	Karbari, local representatives, health workers, teachers, healers and adult community members	Introduce study and identify questions and concerns	Use smaller-group dialogue, record questions and feedback, and adapt explanations to emerging concerns.
Pre-intervention	Special village meetings (20 villages)	Communities with emerging concerns or anticipated participation challenges	Enable focused dialogue and gather feedback	
Pre-intervention	Courtyard/small-group meetings (735; 6,723 adults recorded)	Villagers, karbaris and health workers	Provide smaller-group opportunities for questions and discussion	
Pre-intervention	Poster distribution	Villagers and households	Provide sustained visual reminders in low-literacy settings	Use portable screening and culturally adapted visual communication.
Pre-intervention	Short video sessions (170 sessions; 3,837 participants recorded)	Community members and local health workers	Explain study concepts visually and in local language	
Pre- and during intervention	Basic healthcare alongside the study	Communities across 100 villages	Respond to substantial unmet health needs as part of the community-based study approach	Provide basic care, training and referral; care was offered independently of individual study participation.
During intervention	Media monitoring and rumour response	Local media, social media and community actors	Identify and respond to emerging misinformation	Monitor local information channels and provide timely, consistent clarification.

#### Engage with key community-based stakeholders

Stakeholders offered recommendations based on the study introduction and their experience of the study setting. A recurrent recommendation was to engage key individuals within study villages—including headmen, karbaris, bhantes, Union Parishad (UP) chairmen, and educated youth—early in the process, while recognising that their roles differed across communities. Karbaris were critical throughout the community-based activities.

Some stakeholders highlighted the importance of engaging, educated youth, noting their potential to act as mediators within the community. Sustaining engagement was also considered important, particularly where local actors contributed time to meetings, communication, and follow-up (Table 1).

#### Consider potential participants’ concerns

Stakeholders emphasised the importance of clear operational and safety procedures, including post-vaccination observation and communication about potential adverse events. They also highlighted the need to schedule engagement and intervention activities around religious festivals and jhum cultivation periods.

Food insecurity and poor health conditions were identified as important community concerns. Stakeholders also identified remoteness as a major logistical challenge and recommended coordination with local authorities for access while avoiding communication that could make the study appear to be a developmental activity (Table 1).

#### Address misinformation and accommodate traditional beliefs

Addressing misinformation was considered both sensitive and critical. A recurring concern was that communities might feel that an experimental vaccine was being tested on them. Stakeholders recommended consistent, accurate communication about the vaccine’s regulatory status and prior use. Field team thus used terms such as “WHO recommended” and “WHO prequalified” when describing the vaccine’s regulatory status.

Stakeholders also highlighted the persistent influence of traditional practitioners, which often serve as the first point of contact for health and illness. They recommended that traditional beliefs not be dismissed and that study information be discussed through respectful, culturally sensitive dialogue. This approach was intended to support understanding and informed decision-making rather than to replace community perspectives (Table 1).

#### Provide basic healthcare

Authorities and key stakeholders highlighted the substantial unmet need for basic healthcare in the study setting. In addition to researchers’ prior experience as well, primary healthcare was swiftly incorporated into the community-based study approach, independently of individual participation, alongside the study intervention (Table 1).

Although the initial engagement strategy was informed by stakeholder consultations and formative work, implementation required continuous adaptation. Throughout the trial, engagement evolved in response to community feedback, rumours, seasonal livelihood activities, logistical constraints, and local cultural and religious beliefs. Adaptations included modifying communication approaches, organising additional meetings, strengthening collaboration with trusted local leaders, adjusting implementation schedules, and integrating basic healthcare support where appropriate.

### Lessons from communities and subsequent adaptations during interventions – illustrative case studies

#### Informal dialogue and emerging concerns

Informal platforms provided opportunities to identify community questions and rumours that were not always raised during formal meetings. For example, discussion of the study restriction on participation of pregnant women revealed concerns that the intervention might affect fertility. These concerns were documented as part of the ongoing engagement process.

#### Rumour response and adaptation

Special meetings provided a space to discuss serious rumours and concerns. In one instance, a rumour that the vaccine could cause infertility and death was reported to the field team. The team responded through discussion with community members and, where relevant, through engagement with local media (Table 2).

During festival periods such as Ramadan, community members suggested timings that would avoid disruption of religious practices. The study team adapted the schedule, accordingly, including conducting procedures in the evenings after meals.

In another instance, a rumour associated with a reported dream involving the number 666 was linked by community members to Krama beliefs and interpreted as a sign of inauspiciousness. The case illustrated how concerns about the intervention could intersect with existing religious interpretations (Table 2).

#### Responding to local conflicts affecting engagement

There was a conflict between a karbari’s family and the village health worker’s family, reportedly related to land ownership, which was unrelated to the study but affected engagement. The study team clarified its neutrality and continued dialogue with both parties; participation subsequently resumed.

In another village, after the karbari died, his role and authority passed to his son, but villagers did not recognise his authority. A special meeting was conducted, but the team was unable to secure participation. Other factors may also have contributed to non-participation, illustrating the limits of additional meetings when underlying local power relations and concerns remain unresolved (Table 2).

**Table 2:** Illustrative case studies showing community concerns, engagement responses, adaptations, and observed responses

Case study	Context / barrier	Engagement response / adaptation	Observed response / lesson
Case 1: MVA village (January 2025)	An influential local political actor reportedly disseminated concerns about long-term effects, research ethics, accountability and the focus on Mro communities.	• Direct discussion with the individual; explanation by research physician; invitation for further discussion; community meeting with local leaders; health camp alongside engagement.	Direct opposition appeared to lessen but misinformation continued. Lesson: sustained, multi-level dialogue may be needed when influential actors shape local perceptions.
Case 2: MVDA village (February 2025)	No villagers attended the first intervention round. A rumour linked vaccination with the number “666”, death and religious symbolism, with perceived inconsistencies across villages adding to mistrust.	• Multidisciplinary field visit; discussion with karbari and community members; household engagement; explanation of study purpose and safety; invitation to observe the intervention without obligation.	More than 50 people later came to observe the activities and subsequently decided to participate. Lesson: observation and dialogue can create opportunities for informed reconsideration.
Case 3: MVDA/MVA villages (March 2025)	No villagers attended. Resistance was associated with longstanding rejection of vaccination, spiritual interpretations of a dream, and strong elder influence.	• Repeated field visits and attempts to establish dialogue; meetings with leaders and residents were attempted but access remained restricted.	Vaccination activities were suspended. Lesson: engagement may remain limited where access is restricted and resistance is strongly entrenched.
Case 4: MVDA village (March 2025)	Community members had expressed willingness before intervention, but participation was very limited; local leadership was newly established and had limited authority.	• Pre-intervention discussion with leaders; targeted interpersonal communication; detailed information provided to a local BRAC staff member, with encouragement to independently verify information.	Approximately 80 people were enlisted, but many were ineligible; only a limited number were vaccinated. Lesson: expressed willingness does not necessarily translate into intervention uptake, particularly where local leadership dynamics are changing.
Case 5: MVDA village (March 2025)	A returning migrant worker raised concerns that simultaneous vaccine and drug administration could cause overdose and adverse effects, and left the centre with around 35–40 villagers.	• Immediate discussion at the centre; targeted village visit; multidisciplinary engagement with the individual; community meeting explaining the protocol and co-administration.	Remaining community members returned the next morning and received the intervention. Lesson: informal authority can rapidly shape participation and requires prompt, respectful response.
Case 6: MVDA village (March 2025)	About half the population participated initially; others were concerned about vaccination and possible severe effects.	• Household visits; visual demonstration; invitation to observe the vaccination centre; transport support to reduce access barriers.	Approximately 30 villagers, including the karbari, visited the centre and expressed willingness to receive the intervention. Lesson: direct observation and practical access support can facilitate informed decision-making.
Case 7: Standard-of-care village (March 2025)	No one initially provided DBS. A rumour claimed blood samples would be sent to Bangkok for black magic, compounded by remoteness.	• Same-day field visit; community meeting; explanation of the research purpose and DBS procedure; discussion of the circulating rumour.	Community members participated the following day and provided DBS samples. Lesson: timely rumour identification and direct explanation can address research-related misconceptions.
Case 8: MVA village (April 2025)	20 of 50 expected participants attended in round three. Concerns included mild side effects during earlier rounds, peak jhum cultivation, caregiving demands and misinformation.	• Same-day discussion with karbari and villagers; community meeting; clarification of expected mild reactions; assurance of local health support; vaccination services provided within the village the following day.	The remaining participants were vaccinated the next day. Lesson: adapting timing and location to livelihood demands while providing clear safety information may reduce practical barriers.

Collectively, these illustrative cases show how community engagement evolved rather than operating as a fixed set of activities. Across cases, adaptations included responding to rumours, using trusted local actors, adjusting timing or location, and increasing opportunities for dialogue. The cases also show that engagement did not always resolve concerns or lead to participation.

## Discussion

This implementation case study illustrates stakeholder and community engagement as an adaptive process that evolved throughout a complex malaria elimination trial [27]. The initial strategy was informed by formative research and stakeholder consultation, but implementation required repeated adaptation in response to community feedback, rumours, livelihood priorities, and local cultural contexts [32]. The cases also show that adaptation did not always resolve concerns or result in participation, reinforcing the importance of viewing community engagement as a continuous process rather than a one-time preparatory activity including with definite impacts on participation.

### Emergent challenges related to community engagement and responses

A comprehensive community engagement strategy was developed based on prior experience and formative work [21] to support the first field trial of a malaria vaccine in Asia in a remote and culturally complex setting in rural Bangladesh [12]. The implementation experience revealed both adaptations that were followed by renewed engagement in some settings and situations in which concerns remained unresolved. Because this was a descriptive implementation case study, it was not designed to evaluate the effectiveness of individual engagement activities or to establish causal relationships with participation.

### Remoteness and trust

The study was conducted in remote and isolated villages that were often difficult to access. In some cases, the team was unable to physically reach certain settlements, particularly those located high in the hills. This challenge was even more pronounced for elderly and disabled individuals, limiting their participation in both engagement activities and the study. Poor mobile network coverage and inadequate road infrastructure further constrained communication and access. The remoteness of study sites within the Chittagong Hill Tracts (CHT) has been identified as a persistent challenge for the National Malaria Control Programme as well, affecting access, transportation, and the delivery of health and research activities [33]. These challenges were further compounded by the isolated nature of the villages and their unique social, cultural, political, and historical characteristics.

There was also a deep mistrust of outsiders, shaped by historical experiences such as being deceived on land ownership [13]. This mistrust influenced willingness to engage with the study team, highlighting the need for sustained and adaptive community engagement to build trust and foster relationships with indigenous and marginalised communities [34]. Formative work indicated that village leaders were often important for facilitating engagement; however, in some villages, local political dynamics and interpersonal relationships complicated this process [12].

Although many community members initially expressed willingness during public meetings, a small minority of communities did not participate after implementation began. The reasons were not always clear; field reports suggested that fear of outsiders, concerns regarding informed consent procedures, and vaccine hesitancy may have contributed. The inability to establish the reasons fully is itself an important limitation of the implementation record. This observation echoes findings from several previous studies, many of which reported non-participation but did not examine the underlying reasons for unwillingness to participate [23, 35–41].

### Livelihood and timing constraints

Many community members rely on farming, including shifting cultivation, which requires seasonal mobility and long working hours [23, 42, 43]. This constrained availability for engagement activities and study procedures, particularly during peak cultivation periods.

Concerns about post-vaccination symptoms affecting the ability to work were also reported. Aligning engagement and intervention schedules with local livelihood patterns, rather than expecting communities to adapt to research schedules, was therefore an important practical consideration [44].

### Ethical basis and practical limits of ancillary care

The team provided basic healthcare in response to substantial unmet health needs identified during the community-based study [45]. Conditions such as scabies, diarrhoea, snakebite, and burns were managed. This additional health care services are recognized to be critical, as they form the ethical responsibility for researchers not to overlook the health conditions other than what has been intended in their interventions. It reflects an ethical and practical commitment to avoid conducting a community-based study in isolation from serious, readily treatable health needs [14, 46].

However, the scale of the study across approximately 100 villages made comprehensive ancillary care difficult because staff and resources were limited. The team was not equipped to manage severe or complex conditions, so basic care was combined with referral and periodic medical camps where feasible. This illustrates the practical limits of addressing unmet health needs within large community-based research programmes [47].

Healthcare was offered independently of individual study participation [23, 45, 48]. This was intended to separate care from an obligation to participate and to maintain a clear distinction between ethical responsibilities to the study population and recruitment [45]. Future community-based studies in settings with substantial unmet health needs should consider, prospectively, the ethical basis, scope, referral arrangements, and resource implications of ancillary care.

### Complexity of study design and messaging

The study was a cluster-randomized trial with a factorial design, making it complex to explain, particularly in the context of multiple translations. The multiple intervention arms required distinct messaging [49], and previous research suggests that multiple arms can influence perceptions and acceptability [50, 51].

Neighbouring villages were assigned to different study arms, yet community members interacted in shared spaces such as markets. This led to confusion, comparison between groups, and the spread of rumours. The large and multilingual field team further increased the challenge of maintaining consistent messaging across all engagement activities.

Similar challenges have been observed in malaria studies in Southeast Asia and sub-Saharan Africa, particularly when explaining malaria transmission and infection [14, 25, 52]. These experiences underline the need for flexible communication that can be adapted as misunderstandings emerge during implementation [53].

### Religious and cultural beliefs

Religious and cultural beliefs posed challenges, including concerns about vaccination, blood collection, and possible effects on fertility [9, 54, 55]. Some concerns may have been reinforced by the exclusion of pregnant women from the study.

Traditional belief systems and local understandings of health and illness influenced decision-making, and in some villages the reasons for non-participation were not fully understood despite additional engagement efforts [12]. The cases also revealed complex power relations between community leaders, researchers, and participants, with implications for consent and individual autonomy [56, 57]. Rather than attempting to replace local beliefs through one-way communication, engagement required ongoing dialogue and explanation with trusted local leaders and community members.

### Reflections on what worked, what did not, and why

Several features of the engagement approach appeared useful in supporting implementation. Early involvement of multiple sources of influence, including political authorities, media, NGOs, and the health system, created established channels for communication when rumours or resistance emerged [46, 58, 59]. The cases suggest that responsiveness was most useful when concerns could be discussed through trusted relationships, but this approach was less effective where access was restricted or resistance remained entrenched [60].

The iterative development of visual communication materials allowed complex study concepts to be presented in formats adapted to local literacy levels, languages, and cultural contexts [14]. The local research and engagement team also brought experience in mass drug administration and trial-related engagement, which supported continuity between community relationships and implementation decisions.

The provision of healthcare addressed unmet needs within the study setting and may also have influenced how the research team was perceived [61, 62]. Because care was offered independently of individual study participation, the approach sought to avoid making healthcare contingent on research participation; nevertheless, the potential influence of ancillary care on trust and perceptions of the study should be recognised as part of the implementation context [63].

This synthesis has several limitations. First, it is retrospective and implementer-led, and therefore may be affected by confirmation bias, social-desirability bias, and selective recall or documentation. Routine field records varied in completeness, and the analysis did not include an independent community-led assessment of trust or engagement effectiveness. Second, the eight cases were purposively selected as illustrative examples of variation and were not systematically sampled to represent all programme experiences. Third, translation was incomplete: materials were not produced in all relevant languages, and the site team primarily spoke Bengali rather than all local hill-tribe languages, which may have limited interpretation of some interactions. Fourth, the study was not designed with a comparison group for community engagement and cannot establish causal relationships between engagement activities and participation or other outcomes. Fifth, local leadership structures and the provision of ancillary healthcare were important parts of the implementation context and may themselves have influenced trust, perceptions, and participation, making their effects difficult to disentangle from other factors. Finally, because the implementation team contributed to documentation and interpretation, the findings should be read as a reflective synthesis of practice rather than an independent evaluation. The lessons may be transferable to other remote and underserved settings, but their application requires attention to contextual features such as local governance structures, language, geography, livelihood patterns, and the ethical scope of ancillary care.

## Conclusions

This implementation case study illustrates stakeholder and community engagement as an adaptive process that evolves throughout complex health research. Early stakeholder involvement, responsiveness to community concerns, collaboration with trusted local actors, and flexibility in implementation were recurring features of the approach. The experience also shows that these strategies do not always resolve resistance or produce participation. Although developed within a malaria elimination trial in the Chittagong Hill Tracts, the cases provide practical lessons for community-engaged research in other underserved settings, with transferability depending on local governance, language, geography, livelihood, and health-system context.

## Electronic supplementary material

Below is the link to the electronic supplementary material.

Supplementary Material 1

Supplementary Material 2

Supplementary Material 3

Supplementary Material 4

Supplementary Material 5

## Data Availability

Due to concerns regarding participant confidentiality and the potential risk of identification, the data underlying this study cannot be made publicly available. However, data may be accessed upon reasonable request to the Mahidol Oxford Tropical Medicine Research Unit (MORU) Data Access Committee, in accordance with the Unit’s data access policy (http://www.tropmedres.ac/data-sharing).

## Abbreviations

CHT: Chittagong Hill Tracts
EPI: Expanded Programme on Immunization
FGD: Focus Group Discussion
IDI: In-Depth Interview
KII: Key Informant Interview
MDA: Mass Drug Administration
MVA: Mass Vaccination Administration
MVDA: Mass Vaccination and Drug Administration
RDT: Rapid Diagnostic Test
