# Supplementary material for: Mass vaccination and drug administration for malaria elimination in Bangladesh: design and implementation of a community engagement strategy
Source: Res Involv Engagem. 2026 Oct 29;12:174. doi: 10.1186/s40900-026-00995-1 (PMC13721484; doi:10.1186/s40900-026-00995-1)
Supplement: Supplementary file 1 [file 40900_2026_995_MOESM1_ESM.docx]

**From:** [**GRIPP2 reporting checklists: tools to improve reporting of patient and public involvement in research**](https://link.springer.com/article/10.1186/s40900-017-0062-2)

| **Section and topic** | **Item** | **Reported in revised manuscript** |
| --- | --- | --- |
| 1: Aim | Report the aim of community and stakeholder engagement in the study | Under “Research team organization, roles and training” in the revised manuscript, where the purpose of community and stakeholder engagement is described. |
| 2: Methods | Provide a clear description of the methods used for community and stakeholder engagement in the study | In the Methods section under “Research team organization, roles and training” and “Design, implementation and adaptation of engagement strategies”. The manuscript describes community and stakeholder engagement embedded within the MVDA study; it was not designed as a conventional PPI study. |
| 3: Study results | Report the results of community and stakeholder engagement, including adaptations, responses, and challenges | In the Results section, including the sections describing adaptations, challenges, and illustrative cases. |
| 4: Discussion and conclusions | Comment on how community and stakeholder engagement informed implementation decisions and adaptations, including positive and negative experiences | In the Discussion section, particularly the synthesis of cross-cutting lessons and limitations. |
| 5: Reflections/critical perspective | Comment critically on the engagement experience, reflecting on what worked and what did not, so that others can learn from the experience | In the Discussion section, particularly the synthesis of cross-cutting lessons and limitations. |
