# Supplementary material for: Mass vaccination and drug administration for malaria elimination in Bangladesh: design and implementation of a community engagement strategy
Source: Res Involv Engagem. 2026 Oct 29;12:174. doi: 10.1186/s40900-026-00995-1 (PMC13721484; doi:10.1186/s40900-026-00995-1)
Supplement: Supplementary file 2 [file 40900_2026_995_MOESM2_ESM.docx]

**Timeline of Stakeholder and Community Engagement Activities (MVDA Study)**

**Phase 1: Pre-study Strategy Development (Mar 2023 – Nov 2023)**

**The 2023 activities represent pre-study engagement undertaken before the subsequent trial implementation; they are included to show how the engagement strategy developed over time.**

- **22 Mar 2023** – Workshop 01: Initial communication strategy development (*Dhaka*)
- **Mar–May 2023** – Qualitative research to inform communication strategy (*Lama & Alikadam*)
- **31 May 2023** – Workshop 02: Refinement of strategy based on field findings
- **02 Nov 2023** – Workshop 03: Finalization of communication strategy

*Focus:* Designing culturally appropriate communication approaches and understanding community perceptions.

**Phase 2: Stakeholder Engagement & Tool Development (Mar 2024)**

- **10 Mar 2024** – High-level orientation with policymakers (*Bandarban DC office*)
- **16 Mar 2024** – Workshop 04: Development of communication tools (videos, materials)
- **18 Mar 2024** – Orientation meeting with district stakeholders
- **18 Mar 2024** – Press briefing with journalists

*Focus:* Securing policy support, refining tools, and initiating media engagement.

**Phase 3: Local-Level Engagement & Community Entry (Apr 2024 onward)**

- **01 Apr 2024** – Orientation meeting with village leaders (*Lama*)
- **01 Apr 2024** – Press briefings (*Lama & Alikadam*)
- **From Apr 2024 onward – One-to-one interpersonal communication (4,209 contacts reported)**

*Focus: Community entry, relationship-building, and dissemination of study information.*

**Phase 4: Baseline Assessment & Early Implementation (Jul 2024 – Oct 2024)**

- **05 Jul – 01 Aug 2024** – Census in 100 villages
- **11 Jul 2024 – May 2025** – Medical camps across villages
- **From Jul 2024 (ongoing)** – Healthcare support & referral system
- **Jul – Oct 2024 – Baseline KAP and qualitative study**

*Focus:* Understanding population, addressing immediate health needs, and building trust.

**Phase 5: Coordination & Monitoring Platforms (Jul 2024 – Ongoing)**

- Monthly meetings at:
  - Civil Surgeon Office
  - Zilla Parishad
  - Upazila Health Complex
- Weekly field-level meetings (from Oct 2024)

*Focus:* Continuous coordination, monitoring, and problem-solving.

**Phase 6: Intensive Pre-intervention Engagement and Early Implementation (Oct 2024 – Feb 2025)**

- **Oct 2024 – Feb 2025** – Poster distribution in 100 villages
- **Oct 2024** – 100 village meetings (4,095 participants)
- **Oct 2024 – Feb 2025** –
  - 735 courtyard meetings (6,723 participants)
  - Short video sessions (local language)
- **Oct 2024 – Feb 2025 – Special village meetings (targeting communities where additional engagement was needed)**

*Focus: Broad community engagement, addressing concerns, supporting informed participation, and preparing for implementation.*

**Phase 7: Implementation Support & Risk Communication (Feb 2025 onward)**

- **Feb 2025 – ongoing** – Media monitoring & rumor management
- **Feb 2025 – ongoing** – AE/SAE communication and management
- Ongoing documentation of:
  - Death cases (verbal autopsy)
  - Community feedback & FAQs

*Focus: Safety communication, misinformation response, and ongoing community feedback.*

**Phase 8: Evaluation & Adaptation (2025 – 2026)**

- **Jul 2025** – Poster & flipchart field testing
- **Sep 2025 – ongoing** – Acceptability study (FGDs)
- **Dec 2025** – Special meetings for booster dose hesitancy
- **Nov 2025 – Feb 2026** – Courtyard meetings before booster dose

*Focus:* Assessing acceptability, refining strategies, and supporting booster uptake.
