# Supplementary material for: Mass vaccination and drug administration for malaria elimination in Bangladesh: design and implementation of a community engagement strategy
Source: Res Involv Engagem. 2026 Oct 29;12:174. doi: 10.1186/s40900-026-00995-1 (PMC13721484; doi:10.1186/s40900-026-00995-1)
Supplement: Supplementary file 3 [file 40900_2026_995_MOESM3_ESM.docx]

Community Engagement video

1. <https://youtu.be/nz9bdDr7w0Q>
2. <https://youtu.be/ebYCezvCubo>
3. <https://youtu.be/U22qP_r7MRg>
4. <https://youtu.be/EXbl4wsIfLA>

MVDA Documentary video

1. <https://youtu.be/BwzbkvDZ42M>

Posters

2. <https://drive.google.com/file/d/1FbWF1tZUn894CT6-gInev15K808ADbaD/view?usp=drive_link>
3. <https://drive.google.com/file/d/1MKOMFIEWGwabCuEprBsh_YbEqTkluLIM/view?usp=drive_link>
4. <https://drive.google.com/file/d/1Fzsynf24bUbAz38KQtdNfZfZ652IWW89/view?usp=drive_link>
5. <https://drive.google.com/file/d/1twOQ9wegIt-CgqNHQCV8S3jtkFrvOz-R/view?usp=drive_link>
6. <https://drive.google.com/file/d/1SWt6QPQu4EUAGG9t0Phq6rs3yHkuSfz7/view?usp=drive_link>

Flipchart

1. <https://drive.google.com/file/d/1To5IWUO4grgz6zvutv907UgGki-7zDXZ/view?usp=drive_link>
2. <https://drive.google.com/file/d/1_iAohyVxKPfyxt-HfsOJ0EvTLs_oBtPG/view?usp=drive_link>
3. <https://drive.google.com/file/d/1Z6cJKvUzOkEqlbnqB0bXBcPjDcqyljS3/view?usp=drive_link>
4. <https://drive.google.com/file/d/1Jdcl0l74_x92dJhuTmrJJ9R_B5ywSxFF/view?usp=drive_link>
