# Supplementary material for: Mass vaccination and drug administration for malaria elimination in Bangladesh: design and implementation of a community engagement strategy
Source: Res Involv Engagem. 2026 Oct 29;12:174. doi: 10.1186/s40900-026-00995-1 (PMC13721484; doi:10.1186/s40900-026-00995-1)
Supplement: Supplementary file 4 [file 40900_2026_995_MOESM4_ESM.docx]

**General Concerns About Vaccine & Drug**

**Q: Is there any problem if we do not take the vaccine and drugs?**
A: There is no problem, if you do not take any vaccine or drug. However, taking the vaccine and drugs helps protect you from malaria.

**Q: What is known about the safety of this vaccine?**
A: Yes,the vaccine has undergone safety evaluationand has beenprequalified by the World Health Organization. It has shown about 75% efficacy in preventing malaria.

**Q: Will this vaccine cause death or reduce lifespan?**
A: No, these are rumors.Participants were informed that adverse events can occur and that follow-up and medical support are available. Although there may be some adverse events, such as fever, injection-site pain, and myalgia, these typically resolve within a few days.

**Q: Can this vaccine cause harm or unusual effects (e.g., becoming ghosts)?**
A: No, this is a misconception.The vaccine is intended to prevent malaria; participants were informed about possible adverse events and follow-up procedures. Although there may be some adverse events, such as fever, injection-site pain, and myalgia, these typically resolve within a few days.

**Eligibility & Special Conditions**

**Q: Can pregnant or breastfeeding women take the vaccine or drug?**
A:Pregnant women and women planning pregnancy were not eligible for vaccination in this study. Eligibility of breastfeeding women was determined according to the study protocol and medical assessment.

**Q: Can people with allergies or other illnesses take the vaccine?**
A: Specific medical conditions will be assessed by medical staffs & they will decide whether participants are eligible for vaccination or not. Please consult the MVDA medical team.

**Q: Why are children under 6 months not included?**
A:Children under 6 months of age were not included in this study, in accordance with the study protocol. For these children under 6 months will be excluded from the study.

Q: **Why have you selected only Mro community for the study?**

A: First of all, the villages were selected based on malaria incidence in 2023. The more malaria-prone villages were prioritized. The selected villages include not only the Mro community, but also Tripura, Bengali, Tanchanga, Marma, and Chakma communities.

**Vaccine Administration & Schedule**

**Q: Where will the vaccine be given?**
A: Vaccination will be conducted at designated centers such as local schools or community sites in your village.

**Q: How many doses are required?**
A: A total of four doses: three monthly doses and one booster dose after one year.

**Q: What happens if I miss a dose?**
A: Health workers will follow up and guide you to complete the schedule.

**Side Effects & Safety Monitoring**

**Q: Are there any side effects?**
A:Adverse events can occur.Some people may experience mild symptoms such asfever, myalgia, pain & redness at the injection site etc.

**Q: What should I do if I experience side effects?**
A: Inform the village health worker immediately. The MVDA medical team will provide support and treatment if needed.

**Q: Will medical support be provided if problems occur?**
A: Yes, medical support will be provided, and necessary treatment costs will be covered by the project.

**Q: Could women's reproductive health be impacted?**

**A: Pregnant women and women planning pregnancy were not eligible for vaccination in this study. The study therefore does not provide evidence to determine effects on pregnancy; reproductive-health questions were to be discussed with the study medical team.**

**Effectiveness & Malaria Prevention**

**Q: Can I still get malaria after taking the vaccine and drug?**
A: The vaccine significantly reduces the risk against Plasmodium falciparum & its complications, but it does not guarantee complete protection.

**Q: Is it possible to eliminate malaria through this study?**
A: This study aims to identify effective strategies. Based on results, interventions may be expanded to all villages & it might be a valuable tool for malaria elimination.

**Study Design & Randomization**

**Q: Why are not all villages receiving the vaccine?**
A: Villages are selected through randomization. After evaluation, effective interventions will be provided to control villages.

**Q: What is the standard of care?**
A: It includes use long lasting insecticide treated mosquito nets, environmental cleanliness, Rapid diagnostic tests, and treatment services.

**Blood Collection (DBS & Testing)**

**Q: Why is blood collected?**
A: To test for malaria parasites and monitor infection levels.

**Q: How much blood is taken?**
A: Only a small amount (3–6 drops) from a finger prick.

**Q: Is blood collection harmful?**
A:The procedure involves a small finger-prick blood sample and may cause brief discomfort.Participants were informed about the purpose of blood collection and the study follow-up procedures.

**Q: How often is blood collected?**
A: Every six months total five times for monitoring purposes.

**Q:** Should DBS be collected from everyone or just from malaria patients?

**Drug Use & Participation**

**Q: Should everyone take the drug even if they do not have malaria?**
A: Yes, as part of the study protocol, unless excluded due to specific conditions.

**Q: Can I take other medicines with this vaccine and drug?**
A: Consult a health worker before taking other medications.

**Q: If I get malaria after taking the drug, can I take treatment again?**
A: Yes, standard malaria treatment can still be taken.

**Q:** If someone does not want to take this drug/vaccine in the first round, if he/she wants to take it in the second round, can this drug be given?

**Cost & Access**

**Q: Is there any cost for vaccination or drugs?**
A: No, all services are provided free of cost.

**Q: Will the vaccine and drug be available later in our village(control)?**
A: Yes, based on study results, they may be provided in control villages.

**Community & Trust**

**Q: Who will take responsibility if problems occur?**
A: The MVDA team will take responsibility and provide support.

**Q: Do MVDA workers take the vaccine?**
A: Yes, MVDA workers those who are residing in our randomized villages will also take the vaccine.

**Miscellaneous**

**Q: Are there any food restrictions while taking the drug?**
A: No, you can eat normally.

**Q: What is malaria?**
A: Malaria is a disease caused by parasites transmitted through the bite of infected female Anopheles mosquitoes.

**Q: Why is malaria sometimes not diagnosed by village health workers, but if you go to a doctor and get a malaria test, it is detected? Why is this?**

A: Village health workers perform RDT test to diagnose malaria and sometimes, it shows false negative result. However, when they visit any hospital or doctor, lab team perform both RDT & microscopy which helps to diagnose the false negative cases.

**Q: We would like to meet your senior officers. It would be helpful if they could visit. When will they come?**

A: They will visit frequently in your area in a certain time. When they will visit, we will inform you. Specially they will visit during the campaign.

**Q: Will we get medicine for other diseases?**

A: We will try to organize medical camp on a regular basis & provide you other medical services.
