# Supplementary material for: Mass vaccination and drug administration for malaria elimination in Bangladesh: design and implementation of a community engagement strategy
Source: Res Involv Engagem. 2026 Oct 29;12:174. doi: 10.1186/s40900-026-00995-1 (PMC13721484; doi:10.1186/s40900-026-00995-1)
Supplement: Supplementary file 5 [file 40900_2026_995_MOESM5_ESM.docx]

**MVDA Meeting Files**

**Mass Vaccine and Drug Administration (MVDA), Bangladesh**

# Part A: Strategic Workshops, Orientations, Press Briefings, and Communication Package

# 1. Executive Summary

- Overall purpose: The meetings focused on communication and community engagement for the malaria elimination research titled “Vaccination for All and Medicine for All / Mass Vaccine and Drug Administration (MVDA), Bangladesh.”
- Target area: The project repeatedly identifies Lama and Alikadam upazilas of Bandarban as the main high-risk areas for the research and engagement activities.
- Study structure: The study is described as involving approximately 100 malaria-affected villages/paras, divided into four arms: vaccine plus drug with standard of care, vaccine with standard of care, drug with standard of care, and standard of care only.
- Community engagement: All meetings emphasized that the research cannot succeed without the trust and active involvement of local communities, especially Karbaris, headmen, monks/religious leaders, para workers, women, youth, local government, health workers, and media.
- Communication approach: Messages should be simple, visual, culturally appropriate, locally produced, and delivered in local languages/dialects. Flipcharts and billboards alone are not considered sufficient.
- Main concerns raised: Participants asked about vaccine safety, eligibility, side effects, pregnancy, children below six months, village selection, fairness across arms, malaria testing, treatment access, remote access, and what will happen if malaria is not eliminated in standard-care villages.
- Strategic direction: The strongest recommendation across meetings is to combine scientific transparency with culturally grounded engagement: explain the research, consent, benefits, risks, and full vaccine/drug course clearly before field activities begin.

# 2. Meeting Index

| **No.** | **Meeting** | **Date / Venue** | **Main Focus** |
| --- | --- | --- | --- |
| Workshop 1 | Communication Strategy Development Workshop | 22 March, 2023, CIPRB, Dhaka | Community engagement strategy, study arms, cultural context |
| Workshop 3 | Communication Strategy Workshop / Meeting Minutes | 02 Nov 2023, CIPRB Dhaka | Research objectives, mass vaccine/drug groups, communication materials |
| Workshop 4 | Workshop on Communication Tools | 16 Mar 2024, RSRR Conference Room, Mohakhali | Video, poster, flipchart, upazila awareness meeting decisions |
| Meeting 5 | Orientation Meeting, Bandarban | 10 Mar 2024, DC Office Bandarban | District-level orientation with DC, CS, MP and investigators |
| Meeting 6 | Press Briefing, Bandarban | 18 Mar 2024, CS Office Bandarban | Media briefing and Q&A on MVDA |
| Meeting 7 | Zila Parishad Meeting, Bandarban | 18 Mar 2024, Zila Parishad Bandarban | Local institutional engagement and stakeholder support |
| Meeting 8 | Orientation Meeting, Lama | 01 Apr 2024, UNO Office Lama | Upazila-level orientation and community concerns |
| Meeting 9 | Press Briefing, Lama | 01 Apr 2024, UNO Office Lama | Media briefing and questions from Lama journalists |
| Meeting 10 | Orientation Meeting, Alikadam | 02 Apr 2024, Rupmuhuri Resort | Upazila-level orientation, health department and local stakeholders |
| Meeting 11 | Press Briefing, Alikadam | 01 Apr 2024, Rupmuhuri Resort | Media briefing and technical Q&A |
| Meeting 12 | Village Leaders Orientation | 02 Jul 2024, five key zones of Lama and Alikadam | Karbari/village leader orientation and Q&A |

# 3. Cross-Meeting Key Themes

- Trust-building comes first. Many speakers noted that historical conflict, language barriers, and mistrust make engagement difficult. Trust must be built through respected local leaders and transparent explanation.
- Use “para” rather than “village” where appropriate. The term “para” is more familiar in the Chittagong Hill Tracts context and may improve local understanding.
- Respect local governance. Karbari, headmen, Hill District Council, UNO, UHFPO, Union Parishad representatives, and district administration should be involved before community-level activities.
- Involve women and mothers. Women are influential in family decision-making in indigenous communities and should be directly included in awareness and consent processes.
- Engage youth and local creators. Youth, digital creators, local performers, cultural/sports events, festivals, and offline media can help spread messages in accessible ways.
- Explain research ethics clearly. The consent process, voluntary participation, side effects, benefits, blood collection, eligibility, and reasons for four study arms must be explained simply.
- Use multiple channels. Meetings should combine interpersonal communication, pictorial materials, local-language videos, community clinics, health workers, para workers, religious leaders, and media.
- Clarify that MVDA complements existing care. Standard care—mosquito nets, RDT testing, BRAC/EKOTA health workers, and hospital referral—remains important even where vaccine/drug arms are used.

# 4. Workshop 1 — Communication Strategy Development

Meeting details: 22 March, 2023, CIPRB, Dhaka.

## Summary

Prof. Abul Faiz explained that malaria elimination can involve several methods, with vaccine and drug-based interventions as two major approaches. He emphasized that community engagement is essential and should begin at least one year before implementation.

The proposed study would include villagers aged over six months in about 100 villages of Lama and Alikadam, divided into four study arms. The project period was described as lasting approximately four years, with engagement needed throughout, not only before vaccination.

Dr. Anis Pervez summarized formative discussions in Lama and Alikadam, including sessions with Marma and Mro communities, monks, Karbaris, teachers, youth, settlers, doctors, and NGO workers. Food insecurity, remoteness, inter-ethnic differences, language, culture, and trust were identified as major engagement factors.

Participants recommended using trusted opinion leaders, para workers, monks, religious leaders, youth, mothers, community clinics, traditional healers, pharmacists, security forces, and local government institutions.

## Key points / decisions

- Start communication and engagement at least one year before field implementation.
- Use culturally adapted messages for different ethnic groups and languages.
- Prioritize interpersonal engagement over only posters, billboards, or flipcharts.
- Include women, youth, local leaders, para workers, traditional healers, pharmacists, and local institutions in the engagement plan.
- Consider pictorial communication because many indigenous community members understand visual messages better than text.

# 5. Workshop 3 — Communication Strategy Meeting Minutes

Meeting details: 02 November 2023, CIPRB, Dhaka, Bangladesh.

## Summary

The meeting reviewed the primary research objective: assessing mass vaccination, mass drug administration, and standard of care in malaria-prone communities.

Group discussions focused on WHO malaria elimination goals, Bangladesh Government initiatives, vulnerable populations, malaria transmission, eligibility, consent, side effects, benefits, full course completion, and how to motivate participation.

Another discussion focused on community culture, entertainment, content language, local dialects, potential actors or celebrities, and communication materials that could reach local people.

## Key points / decisions

- Communication materials must explain who can participate and who cannot.
- Messages must disclose potential side effects of drugs and vaccines.
- The full course of vaccine/drug must be emphasized.
- Local dialect, cultural practices, and entertainment patterns should shape content design.
- A project slogan or key phrase should be selected after testing ideas with communities.

# 6. Workshop 4 — Communication Tools Review

Meeting details: 16 March 2024, 10:00 AM–12:45 PM, CIPRB Conference Room, Mohakhali, Dhaka.

## Summary

Four videos developed for community engagement were reviewed. The meeting focused on making communication materials clearer, more accurate, more inclusive, and more motivating for community participation.

The group discussed video edits, poster size and lamination, flipcharts, upazila-level awareness meetings, post-Eid cultural/sports activities, medical camps, and final coordination.

## Key points / decisions

- Remove all tobacco-related images and replace them with jungle or suitable alternative visuals if needed.
- Add Bengali subtitles and ensure both male and female voice-over narration.
- Include all age groups, health complexes, community clinics, medical and vaccine-related visuals.
- Show malaria symptoms separately, one symptom per frame.
- Add consent statements at the beginning or end of the videos.
- Improve visual clarity and increase font size where possible.
- Finalize poster size and print specifications; laminate after printing rather than printing directly on laminated material.
- Arrange upazila-level awareness meetings in Lama and Alikadam with entrepreneurs, headmen, health workers, UNO/UHFPO/Chairman coordination, food, and honorarium budgets.

# 7. Orientation Meeting — Bandarban

Meeting details: 10 March 2024, DC Office, Bandarban. Participants included DC, DCS, CS, MP, and MVDA investigators.

## Summary

The Civil Surgeon opened by describing the government commitment to eliminate malaria by 2030 and noted that Bandarban carried a large burden of malaria, especially Lama and Alikadam. He described the meeting as an orientation on “Vaccination for All and Medicine for All.”

Prof. Abul Faiz explained the current standard of care: insecticide-treated nets, testing and treatment by health workers, and hospital care for severe malaria. He stated that combining standard care with vaccine and medicine could accelerate elimination.

The study was described as covering 100 selected high-risk villages, randomized into four groups: standard care only, standard care plus vaccine and drug, standard care plus drug, and standard care plus vaccine. Eligible participants were residents older than six months who consented. Pregnant women, women planning pregnancy, people with severe illness, and those with previous serious vaccine reaction were excluded.

NMEP representatives said malaria had declined nationally but remained concentrated in the hill districts. They argued that standard care alone may not be enough to meet the 2030 elimination target.

Local leaders and speakers emphasized awareness, stakeholder cooperation, local governance, safety monitoring, and involving public representatives. The MP shared personal experience with malaria and stressed that awareness is the biggest treatment in communities where traditional healing and delayed care remain common.

## Key points / decisions

- Conduct broader orientation meetings in Lama and Alikadam with Karbaris, headmen, and public representatives.
- Explain side effects clearly and consider observation after vaccination, similar to COVID vaccination practice.
- Involve local representatives and administrative structures before and during field activities.
- Identify & treat their health if possible
- Train and use community-level workers and youth to reach remote paras and explain proper treatment-seeking.

# 8. Press Briefing — Bandarban

Meeting details: 18 March 2024, CS Office, Bandarban. Participants included CS, DCS, journalists, and MVDA investigators.

## Summary

The briefing introduced the MVDA research to local journalists and explained its link to the national malaria elimination target. The project was presented as an intervention-oriented research activity under the leadership of Prof. Abul Faiz with national and international partners.

Journalists asked practical questions about whether the vaccine is for healthy or sick people, whether malaria testing will be done first, how the different study arms will show which approach is effective, whether vaccine recipients still need medicine, side effects, pregnancy, public updates, remote villages, selection of communities, and whether the project will be seen as experimental.

The investigators explained that vaccines are given to healthy people, current fever and health status will be checked, the four-arm design is needed to compare effects, and the research team will share updates periodically. Questions about pregnancy and children below six months required careful protocol-based answers.

## Key points / decisions

- Prepare clear public-facing answers for common media questions on safety, eligibility, side effects, malaria testing, village selection, and study arms.
- Provide periodic updates to media as implementation and data collection progress.
- Use journalists as communication partners, not only as observers.

# 9. Zila Parishad Meeting — Bandarban

Meeting details: 18 March 2024, Zila Parishad, Bandarban. Participants included DCS, CS, Chairman Zila Parishad, and MVDA investigators.

## Summary

The meeting focused on building institutional support from the Hill District/Zila Parishad structure. Speakers welcomed the research and emphasized that reaching remote paras would require local governance cooperation.

Participants asked how the strategy was planned, whether field teams would actually reach remote areas, how Karbaris and local leaders would be involved, and whether results would be returned to the community.

The research team responded that Karbaris would be included in the plan, and that sharing research results with the local area is part of responsible research practice.

## Key points / decisions

- Coordinate with the Zila Parishad and relevant local bodies.
- Work with education officers and other departments where local networks can support awareness.
- Identify & treat their health if possible
- Involve local leaders especially karbari
- Ensure research findings are communicated back to the study area.

# 10. Orientation Meeting — Lama

Meeting details: 01 April 2024, UNO Office, Lama, Bandarban. Participants included UNO, DCS, CS, UHFPO, Upazila Parishad representatives, UP members/chairmen, and investigators.

## Summary

The Lama orientation explained the research to upazila-level leaders and community representatives. Speakers described malaria control successes through mosquito nets, testing, treatment, and health education, while noting that elimination by 2030 may require additional methods such as vaccine and medicine.

Community representatives raised practical concerns: how to address concerns people who do not want to take medicine, why people should trust a new vaccine, whether previous malaria work had already helped, how mosquito net distribution could be improved, and how long vaccine protection may last.

The research team emphasized that this is a research program requiring consent and continuous explanation; community meetings and village-level communication would follow.

## Key points / decisions

- Use local meetings and village-level engagement to explain why medicine and vaccine may be needed.
- Address trust concerns by explaining prior vaccine use, WHO prequalification context, and known safety information.
- Identify & treat their health if possible
- Improve coordination around mosquito net distribution concerns and local prevention practices.
- Clarify vaccine duration, dosing schedule, and follow-up in simple language.

# 11. Press Briefing — Lama

Meeting details: 01 April 2024, UNO Office, Lama, Bandarban. Participants included UNO, DCS, CS, UHFPO, Upazila Parishad representatives, journalists, and investigators.

## Summary

Prof. Abul Faiz explained that the research is conducted under the national malaria elimination program with support from Dev Care Foundation, BRAC, EKOTA, and Mahidol Oxford Research Unit. He described malaria symptoms, standard care, the high-risk nature of Lama and Alikadam, and the plan to work in 100 villages with about 10,000 people.

Journalists asked about malaria species in Bangladesh, whether the communication video could be shared, whether more symptoms should be added to the video, why the research is only in Lama and Alikadam, whether the R21/Matrix vaccine had been used elsewhere, what happens after the research period, timing of net distribution, and whether the vaccine is used in other countries.

The team responded that Bangladesh mainly has falciparum and vivax malaria, the selected villages are based on 2023 case burden, the video is for community engagement and may be improved, and successful methods could be expanded later.

## Key points / decisions

- Share appropriate communication materials with journalists when finalized.
- Consider adding additional symptoms or clearer images to the video if feasible.
- Explain village selection criteria based on malaria burden.
- Clarify that the vaccine is part of a research protocol and, if successful, may inform broader use.

# 12. Orientation Meeting — Alikadam

Meeting details: 02 April 2024, Rupmuhuri Resort. Participants included CS, DCS, UNO, BGB, UHFPO, Chairman, and investigators.

## Summary

The UHFPO opened by explaining that existing malaria services include insecticide-treated nets, health education, and treatment. These services have reduced malaria, but elimination by 2030 requires stronger methods. The MVDA research was introduced as one such approach for Lama and Alikadam.

Dr. Mushfiqur Rahman presented the research protocol and collaborating organizations, including national malaria authorities, Chittagong Medical College, Dev Care Foundation, BRAC, CIPRB, MORU Bangkok, and Jenner Institute, Oxford University. He explained malaria transmission through female Anopheles mosquitoes, symptoms, severe disease risk, existing prevention, and the need for vaccine/drug trials in high-burden areas.

Speakers highlighted that malaria has decreased compared with the past, but Alikadam still has a significant burden. Local cooperation, mapping, village selection, health worker engagement, and community understanding are crucial.

## Key points / decisions

- Continue explaining the research protocol to local leaders and community groups.
- Use local health workers and stakeholders to support recruitment, consent, follow-up, and awareness.
- Make the relationship between existing malaria services and the MVDA research clear.

# 13. Press Briefing — Alikadam

Meeting details: 01 April 2024, Rupmuhuri Resort, Alikadam. Participants included DCS, CS, UHFPO, journalists, and investigators.

## Summary

The Alikadam press briefing introduced the MVDA project and explained why Bandarban, especially Lama and Alikadam, is central to the malaria elimination challenge. Prof. Abul Faiz described the current standard care package and the challenge of reaching zero malaria by 2027 and sustaining it until 2030.

Journalists asked whether multiple malaria species exist, why malaria decreased and then increased again, how the 100 villages will be divided, how hard-to-reach areas will be covered, whether teak trees are linked with mosquitoes, whether field workers will be vaccinated, whether there is a mosquito-killing program for dengue, and how the project will know whether vaccine or drug is effective.

The research team explained that vector behavior and parasite survival are complex, malaria risk relates to breeding places and forest/jhum exposure, the research targets parasites rather than mosquito killing, and the four-group design is needed to compare vaccine, drug, combined, and standard-care approaches.

## Key points / decisions

- Prepare a simple explanation of the four-arm study design for the public and media.
- Clarify that MVDA is research focused on malaria parasites, not a dengue or mosquito-eradication project.
- Explain hard-to-reach coverage through community empowerment and one volunteer per village/para where applicable.
- Continue to connect journalists with updated messages and findings.

# 15. Consolidated Action Matrix

| **Area** | **Action Required** | **Responsible / Lead** |
| --- | --- | --- |
| Community entry | Obtain support from Karbaris, headmen, monks/religious leaders, para workers, local government, Hill District Council/Zila Parishad, UNO/UHFPO, and public representatives. | Engagement team + field team |
| Message testing | Pre-test slogans, videos, pictorial messages, and explanations in local languages/dialects before full use. | Communication team + anthropologists |
| Video finalization | Add subtitles, improve font/visual clarity, include community clinics, health complexes, vaccine visuals, consent messages, symptom frames, and both male/female voice-over. | Producer + Investigators |
| Media engagement | Prepare FAQs for journalists and provide periodic updates on implementation, data collection, and research progress. | Project leadership + communication team |
| Community meetings | Hold upazila and para-level awareness meetings before intervention; schedule with UNO/UHFPO/Chairman and local leaders. | Field coordination team |
| Consent and ethics | Explain voluntary participation, eligibility, exclusions, side effects, blood collection, confidentiality, and the four-arm design in simple terms. | Investigators + engagement field assistants |
| Women and youth involvement | Include mothers, women leaders, youth, digital creators, and local performers in message delivery. | Community engagement team |
| Village health workers | Recruit/train village health workers and clarify their roles in follow-up, missed doses, fever referral, and community questions. | Engagement coordinator + group leaders |
| Hard-to-reach coverage | Plan access for remote hills/paras, market-day communication, and offline digital content where internet is limited. | Field operations team |
| Follow-up and safety | Observe participants after vaccination where appropriate and maintain clear medical follow-up/referral pathways for adverse events or fever. | Medical team + MVDA staff |

# 16. Consolidated FAQ for Community and Media Use

| **Question** | **answer** |
| --- | --- |
| Who will receive the vaccine? | Healthy eligible residents above six months who live in selected villages/paras and give informed consent, subject to the research protocol. |
| Will sick people receive the vaccine? | Vaccines are generally given to healthy people. Current fever or illness should be checked before vaccination. |
| Will malaria testing be done first? | Health status and fever will be checked; RDT testing remains important for suspected malaria. The research protocol will define testing for study measurements. |
| Why are there four groups? | The four arms allow researchers to compare vaccine, drug, combined vaccine-plus-drug, and standard-care approaches. |
| Why not all villages? | Villages/paras are selected based on malaria burden and research design; selection and allocation are described as lottery/randomization-based. |
| What if someone misses vaccination day? | They should inform the MVDA village health worker so the team can decide how to manage the missed dose according to schedule and protocol. |
| How many vaccine doses are planned? | The village-leader Q&A section states three doses one month apart, plus a booster after one year. |
| What about pregnant women or children under six months? | These groups require protocol-based answers; the source notes that pregnant women and children below six months should not be included unless the research team/protocol allows. |
| What if side effects occur? | Mild vaccine-type reactions such as pain, redness, swelling, or mild fever may occur; the medical team should provide follow-up and referral if needed. |
| Where should malaria treatment be taken now? | Existing malaria services should continue: BRAC/EKOTA/health workers for testing and treatment, and hospital referral for severe malaria. |
| Does this project kill mosquitoes? | The MVDA research focuses on malaria parasites and protection/treatment. Mosquito nets, cleanliness, and bite prevention remain essential. |
| Who benefits? | Eligible consenting residents of selected villages/paras may benefit directly, and if the intervention proves successful it may guide wider malaria elimination strategies. |

# 17. Recommended Communication Package

- A one-page pictorial leaflet explaining malaria symptoms, prevention, MVDA purpose, eligibility, doses, consent, and fever referral.
- A short local-language video with subtitles, clear visuals, symptom frames, health facility images, and consent message.
- A field worker flipchart with simple illustrations and scripted answers for common questions.
- Separate FAQ sheets for journalists, Karbaris/headmen, mothers/women groups, and youth volunteers.
- Community meeting scripts for upazila, para, market-day, festival, and evening sessions.
- Training guide for village health workers and engagement field assistants.
- Media update template for periodic briefings and press releases.

# Part B: Field and Community Meeting Reports

This part is compiled from the detailed meeting-report file. It preserves the village-level model meetings, Karbari meetings, special meetings before intervention, meetings during intervention, booster-preparation meetings, consolidated questions/responses, and observations.

1. Part 1: Village-Level Model Meeting Reports
2. Part 2: Meetings with Karbaris
3. Part 3: Special Meetings Before Intervention
4. Part 4: Special Meetings During Intervention
5. Part 5: Special Meetings Before Booster Vaccination

# Part 1: Village-Level Model Meeting Reports

Compiled Meeting Reports

Mass Vaccine & Drug Administration (MVDA), Bangladesh

Community Engagement / Village-Level Meeting Reports

# Contents

6. 1. Consolidated Meeting Register
7. 2. Common Meeting Agenda and Engagement Flow
8. 3. Common Field Challenges
9. 4. Detailed Structured Meeting Reports
10. 5. Consolidated Community Questions and Responses

# 1. Consolidated Meeting Register

| **No.** | **Village/Para** | **Zone / Upazila** | **Group** | **Date** | **Time** | **Attendance** | **Venue** | **Reported by** |
| --- | --- | --- | --- | --- | --- | --- | --- | --- |
| 1 | Jira Para | Kurukpata Zone, Alikadam | Current ongoing service | 14 Oct 2024, Monday | 9:30 AM-11:15 AM | 70 reported; male 36, female 35 | One house in Jira Para | Lipi Tripura |
| 2 | Janali Bazar Para | Kurukpata Zone, Alikadam | Medicine for All | 14 Oct 2024, Monday | 4:30 PM-7:00 PM | 36 reported; male 19, female 17. Narrative also mentions 46 including children. | Courtyard of Abu Salek's house | Kraingkong Mro |
| 3 | Mering Char Para | Kurukpata Zone, Alikadam | Vaccine for All | 14 Oct 2024, Monday | 6:30 PM-8:00 PM | 75 reported; male 33, female 28 | Para chief's courtyard | Moni Mukul Chakma |
| 4 | Bidyamoni Para | Kurukpata Zone, Alikadam | Vaccine and Medicine for All | 14 Oct 2024, Monday | 4:30 PM-7:00 PM | 50 total; male 25, female 25 | Open field in the middle of the para | Chathowai Marma |
| 5 | Chingkum Mro Para | Lama, Bandarban | Vaccine and Medicine for All | 08 Oct 2024, Tuesday | 6:45 PM-8:10 PM | 76 total; male 24, female 37, children 15. Brief form also states 76; male 29, female 47. | Buddhist temple/Kiang | Moni Mukul Chakma |
| 6 | Gila Para | Taopara Zone, Lama | Current ongoing service | 13 Nov 2024 | 6:00 PM-8:00 PM | 118 total | Church in Gila Para | Moni Mukul Chakma |
| 7 | Kompong Para | Borkola Jhiri Zone, Lama, Bandarban | Project awareness / Medicine for All | 08 Oct 2024, Tuesday | 12:00 PM start; medical camp from around 2:40 PM | 42 total in narrative. Brief form states 27 adults plus staff. | Karbari's courtyard | Kraingkong Mro |
| 8 | Chhalom Para | Borkola Jhiri Zone, Rupsi Para, Lama | Current ongoing service | 08 Oct 2024, Tuesday | 12:30 PM-3:00 PM | 50 participants; female 30, male 20. Total present with dignitaries: 55. | Chongnak Mro's house | Mrs. Lipi Tripura |
| 9 | Chingkum Mro Para (brief village-ID record) | Borkola Jhiri Zone, Lama | Vaccine for All | 08 Oct 2024, Tuesday | Not specified in brief table | 76 total; male 29, female 47 | Kiang, Chingkum Mro Para | Moni Mukul Chakma |
| 10 | Chotkola Jhiri | Borkola Jhiri Zone, Lama | Vaccine and Medicine for All | 08 Oct 2024, Tuesday | Not specified in brief table | 48 total; male 23, female 25 | Kiang, Chingkum Mro Para | Chathowai Marma |
| 11 | Kompong Para (brief village-ID record) | Borkola Jhiri Zone, Lama | Medicine for All | 08 Oct 2024, Tuesday | Not specified in brief table | 27 reported; male 19, female 9 | Karbari's courtyard | Kraikshong Mro |
| 12 | Chhalom Para (brief village-ID record) | Borkola Jhiri Zone, Lama | Current ongoing service | 08 Oct 2024, Tuesday | Not specified in brief table | 50 total; male 30, female 20 | Karbari's courtyard | Lipi Tripura |

# 2. Common Meeting Agenda and Engagement Flow

- Registration, greetings, and seating of participants and invited guests.
- Introduction of Dev Care Foundation (MVDA) staff, MVDA para health workers, local NGO workers, community leaders, religious leaders, teachers, and other participants.
- Welcome speech by the MVDA para health worker or meeting facilitator.
- Presentation on the MVDA research using posters and flipcharts.
- Explanation of the relevant study group: Current Ongoing Service, Vaccine for All, Medicine for All, or Vaccine and Medicine for All.
- Discussion of eligibility, consent, follow-up, health care support, blood collection/DBS, vaccine/medicine arrangements, and possible side effects.
- Collection of community feedback and community-level agreement or permission.
- Question-and-answer session.
- MVDA video screening by projector where possible.
- Closing remarks by the para chief/karbari, religious leader, ward member, teacher, or other local leader.
- Snacks, meals, and/or medical camp where applicable.

# 3. Common Field Challenges

- During the jhum farming/work season, many residents are away during the day, making daytime attendance difficult.
- Meetings often need to be held in the evening, but lighting and electricity are limited in several paras.
- Mobile network coverage is weak or unavailable; staff sometimes need to climb to higher ground to find signal.
- Travel is difficult; some paras require a combination of vehicle, boat, and 30-60 minutes of walking.
- Residents are often tired after returning from work, so some are reluctant to attend evening meetings.
- Some locations face clean water and sanitation challenges for field teams.
- In some meetings, participants were initially quiet and hesitant to ask questions or share opinions.
- Community members suggested more follow-up meetings and evening schedules, and requested video screening on a larger screen where possible.

# 4. Detailed Structured Meeting Reports

## 1. Jira Para - Current Ongoing Service

### Meeting Details

| **Village/Area** | **Jira Para, Kurukpata Zone, Alikadam** |
| --- | --- |
| Date and Time | 14 October 2024, Monday; 9:30 AM-11:15 AM |
| Group | Current Ongoing Service |
| Participants | Dev Care Foundation engagement coordinator, research physician, engagement group leader, medical assistant, field assistant, para health worker, BRAC worker, para chief, and community residents. Total reported: 70 participants (male 36, female 35). |
| Venue | A relatively large house in Jira Para. A larger open place could not be found after walking around the para. |
| Purpose | To conduct a model village-level meeting and inform residents about the MVDA research. |
| Reported by | Lipi Tripura, Engagement Group Leader, Dev Care Foundation, Lama |

### Agenda / Topics Discussed

- Introductions between community members and Dev Care Foundation staff.
- Welcome speech by the MVDA para health worker.
- Discussion on malaria research led by the group leader.
- Flipchart presentation on the Current Ongoing Service group.
- Collection of community feedback.
- MVDA video screening by projector.
- Closing remarks by the para chief/karbari and light snacks.

### Activities / Proceedings

The meeting time and venue were fixed the previous day in consultation with the para chief and MVDA para health worker. The para had 40 households. The para health worker visited each household and invited two adults from every family. Games were not organized because there was no suitable environment for them. On the advice of the karbari, the meeting was held in the morning because many villagers would later leave for jhum work and stay overnight in the fields.

### Questions and Responses

| **Question / Concern** | **Response / Explanation** |
| --- | --- |
| If someone becomes ill after joining the research, will you pay for treatment? | Yes. If any health problem occurs after participation, continuous follow-up and treatment will be arranged. Dev Care Foundation will bear the necessary treatment costs. |
| Why will blood be taken every six months? | The purpose is to test, using advanced machines, whether malaria parasites are present in the blood. |
| Will the malaria vaccine later be given in our para as well? | After the effectiveness of the malaria vaccine is assessed, the intervention that shows good results may be provided in your para later. |

## 2. Janali Bazar Para - Medicine for All

### Meeting Details

| **Village/Area** | **Janali Bazar Para, Kurukpata Zone, Alikadam; venue noted as Janali Bazar Bangali Para, Alikadam, Bandarban** |
| --- | --- |
| Date and Time | 14 October 2024, Monday; 4:30 PM-7:00 PM |
| Group | Medicine for All |
| Participants | Dev Care Foundation engagement coordinator, group leader, medical assistant, field assistant, para health worker, local NGO/Ekta worker, mosque imam, para chief, and community residents. Total reported: 36 (male 19, female 17). The narrative also mentions 46 people including men, women, and children. |
| Venue | Courtyard of Abu Salek's house, the younger brother of the village chief/karbari. |
| Purpose | To conduct a model village-level meeting and inform residents about the malaria elimination health research. |
| Reported by | Kraingkong Mro, Engagement Group Leader, Dev Care Foundation, Lama |

### Agenda / Topics Discussed

- Staff and community introduction.
- Welcome speech and malaria research discussion.
- Flipchart presentation on Vaccine for All and Medicine for All.
- MVDA video screening and collection of community feedback.
- Speeches by local NGO worker, mosque imam, and para chief/karbari.
- Food served at the end.

### Activities / Proceedings

The team contacted the para chief before the meeting. The para had 17 households, and two adults from each family were invited. On the morning of 14 October 2024, Kraingkong Mro and Medical Assistant Manjuk Chakma purchased and arranged food supplies. The team left for the para at about 1:30 PM and arrived around 2:30 PM. The meeting began at 4:30 PM in Abu Salek's courtyard. The imam chaired the meeting; because the imam was present, residents expressed agreement through him to receive malaria medicine. A projector video was shown and the meeting ended around 7:00 PM with remarks from the imam and karbari.

### Questions and Responses

| **Question / Concern** | **Response / Explanation** |
| --- | --- |
| Where will malaria medicine be provided? | Medicine will be provided to residents of the para at Janali Para Government Primary School. |
| Who will take responsibility if someone has a problem after taking the malaria medicine? | The medicine is not expected to have significant side effects. If any problem occurs after taking it, residents should contact Para Health Worker Anjali Tripura. |

## 3. Mering Char Para - Vaccine for All

### Meeting Details

| **Village/Area** | **Mering Char Para, Kurukpata Zone, Alikadam** |
| --- | --- |
| Date and Time | 14 October 2024, Monday; 6:30 PM-8:00 PM |
| Group | Vaccine for All |
| Participants | Research physician, engagement group leader, medical assistant, field assistant, para health worker, religious leader, school teacher, ward member, BRAC and Ekta workers, para chief, and community residents. Total reported: 75 (male 33, female 28). |
| Venue | Courtyard/compound of the para chief's house, selected because it was spacious and had solar lighting. |
| Purpose | To conduct a model village-level meeting and inform residents about the malaria elimination health research. |
| Reported by | Moni Mukul Chakma, Engagement Group Leader, Dev Care Foundation, Lama |

### Agenda / Topics Discussed

- Introductions and welcome speech by the MVDA para health worker.
- Malaria research discussion by the group leader.
- Flipchart presentation on the Vaccine for All group, conducted in the local Mro language for better understanding.
- Collection of community feedback and MVDA video screening.
- Remarks by the para chief, school teacher, and ward member.
- Thanks from Dev Care and food service.

### Activities / Proceedings

The team coordinated with the para chief and MVDA para health worker the previous day. The para had 47 households, and two adults from each family were invited. Because most adults returned tired from work, only children were available for games in the afternoon. The team organized rooster-fight games and a running competition for young boys; notebooks and pens were given as prizes to the winners.

### Questions and Responses

| **Question / Concern** | **Response / Explanation** |
| --- | --- |
| Why will the malaria vaccine not be given in nearby Marma and Chakma villages? Why only in the Mro para? | Local NGO BRAC and Ekta reported that more than five malaria cases had been identified in this Mro para within the past year. Therefore, this village was selected for the vaccine group. Nearby paras had fewer detected cases and were not included in the research group. |

## 4. Bidyamoni Para - Vaccine and Medicine for All

### Meeting Details

| **Village/Area** | **Bidyamoni Para, Kurukpata Zone, Alikadam** |
| --- | --- |
| Date and Time | 14 October 2024; 4:30 PM-7:00 PM |
| Group | Vaccine and Medicine for All |
| Participants | 42 community participants plus five engagement field assistants, one MVDA para health worker, one engagement group leader, and one medical assistant. Total: 50 (male 25, female 25). |
| Venue | Large open field in the middle of Bidyamoni Para, beside the Matamuhuri River area. |
| Purpose | To present the project at para level, explain selection and eligibility, and obtain community-level agreement or permission for participation. |
| Reported by | Chathowai Marma, Engagement Group Leader, MVDA, Lama-Bandarban |

### Agenda / Topics Discussed

- Registration and exchange of greetings.
- Seating of the chairperson and invited guests.
- Welcome remarks and introductions.
- Project presentation through printed flipcharts on Vaccine for All and Medicine for All.
- Collection of feedback and question-and-answer session.
- Thanks, chairperson's remarks, community-level agreement or permission from residents, and closure.

### Activities / Proceedings

The group leader, medical assistant, and five engagement field assistants visited Bidyamoni Para. They walked around the para, invited residents to attend, followed up with the MVDA para health worker, selected the meeting place, and prepared seating. As villagers arrived, the team greeted them and guided them to sit. The meeting was completed on time and participants had food together before leaving around 8:00 PM.

### Questions and Responses

| **Question / Concern** | **Response / Explanation** |
| --- | --- |
| Who will receive this vaccine? | Everyone older than six months may receive it if they are not seriously ill, do not plan to have a child within the next year, and provide consent. |
| Will you come to the para to provide the vaccine? | At present there is no plan to provide the vaccine and medicine inside the para. The sub-zone selected for this para is Krongleng Para School. If needed, the team may later consider providing services in the para. |
| Can you change the sub-zone and provide the vaccine/medicine in a nearby area? | There is currently no plan to change the sub-zone. Since residents said Krongleng Para is far and Jira Para would be better, the team will discuss the issue with the authority and inform the community later. |

## 5. Chingkum Mro Para - Vaccine and Medicine for All

### Meeting Details

| **Village/Area** | **Chingkum Mro Para, Lama, Bandarban** |
| --- | --- |
| Date and Time | 08 October 2024, Tuesday; 6:45 PM-8:10 PM |
| Group | Vaccine and Medicine for All |
| Participants | Engagement coordinator, group leader, medical assistant, field assistant, para health worker, local NGO BRAC and Ekta workers, local Buddhist monk, para chief, and community residents. Total: 76 including Dev Care staff (male 24, female 37, children 15). |
| Chairperson | Para chief/karbari - Mangya Mro |
| Venue | Chingkum Mro Para; meeting held inside the Buddhist temple/monastery after permission from the monk. |
| Purpose | To conduct a model meeting and inform residents about the malaria elimination research. |
| Reported by | Moni Mukul Chakma, Engagement Group Leader, Dev Care Foundation |

### Agenda / Topics Discussed

- Introduction of Dev Care Foundation staff and welcome remarks.
- Discussion on malaria research and presentation through flipchart.
- Collection of community feedback and MVDA video screening.
- Speeches by local NGO worker, local Buddhist monk, and para chief/karbari.
- Dinner served.

### Activities / Proceedings

The team contacted the para chief in advance. The para had 44 households, and two adults from each family were invited. On 8 October 2024, the team purchased dinner supplies in the morning, left Lama office at about 3:30 PM, and reached the para around 4:20 PM. Few residents were present at first because most had not yet returned from jhum cultivation. Staff and available residents helped prepare food. As residents returned from jhum work, they assembled at the meeting venue. The meeting was chaired by the para chief. Residents listened attentively, asked questions, and the team responded. A video in the local Mro language was shown on a large screen; participants said they understood it well.

### Questions and Responses

| **Question / Concern** | **Response / Explanation** |
| --- | --- |
| When and where will the malaria vaccine be given? | The source records the question but does not provide a detailed written answer in this section. |
| Who will take responsibility if someone has a problem after taking the malaria vaccine? | The team indicated that staff would respond to health concerns and keep contact with residents. |
| If one family member receives the vaccine, do the other family members also need to receive it? | The source records the question but does not provide a detailed written answer in this section. |

### Challenges / Observations

- It was work season, so few people were available in the para during the daytime.
- Starting the meeting at the scheduled time was difficult.
- Mobile network was unavailable except on higher hills.
- Evening meetings faced lighting problems.
- Residents were tired after jhum work and some did not want to attend in the evening.

## 6. Gila Para - Current Ongoing Service

### Meeting Details

| **Village/Area** | **Gila Para, Taopara Zone, Lama** |
| --- | --- |
| Date and Time | 13 November 2024; 6:00 PM-8:00 PM |
| Group | Current ongoing service |
| Participants | Religious leader, Dev Care Foundation engagement group leader, medical assistant, field assistant, para health worker, BRAC worker, para chief, and community residents. Total reported: 118. |
| Venue | The church in Gila Para, where local residents usually hold meetings. Solar electricity was available, and the meeting was held with permission from the karbari and community. |
| Purpose | To conduct a model village-level meeting and inform residents about the malaria elimination health research. |
| Reported by | Moni Mukul Chakma, Engagement Group Leader, Dev Care Foundation, Lama |

### Agenda / Topics Discussed

- Introductions and welcome remarks by the MVDA para health worker.
- Malaria research discussion by the group leader.
- Flipchart presentation on the Vaccine for All topic.
- Collection of community feedback and MVDA video screening.
- Remarks by the religious leader/pastor and para chief/karbari.
- Dinner served.

### Activities / Proceedings

Gila Para is a remote village in Taopara Zone, about three hours on foot from Taopara. Before the meeting, the para karbari was contacted and informed of the reason for gathering the community. Two guardians from each household were invited with the karbari. The discussion was presented using posters. Rinthara Tripura, engagement field assistant, explained the content in the Tripura language. Residents listened attentively and provided community-level agreement or permission to participate in the research. The para chief declared the meeting closed.

### Questions and Responses

| **Question / Concern** | **Response / Explanation** |
| --- | --- |
| Many people had problems after taking the coronavirus vaccine. Will there be problems if we take this vaccine? | This vaccine is not the same as the coronavirus vaccine. It has been stated to be 77% safe. If a health problem occurs after research participation, continuous care and treatment will be arranged, and Dev Care Foundation will bear necessary treatment costs. |
| Can people with allergies receive the vaccine? | If immediate treatment is needed, the person will not be vaccinated at that time. A person with long-term allergy or another chronic condition may receive the vaccine if otherwise suitable. |
| Will young children receive the vaccine? | Children below six months, breastfeeding mothers, and pregnant women will not receive this vaccine. Children of other eligible ages may receive it. |
| What if someone becomes pregnant after receiving the first vaccine dose? | She will not receive the next vaccine dose and will be kept under continuous observation. |

## 7. Kompong Para - Village-Level Project Meeting and Medical Camp

### Meeting Details

| **Village/Area** | **Kompong Para, Borkola Jhiri Zone, Lama, Bandarban** |
| --- | --- |
| Date and Time | 08 October 2024; meeting began around 12:00 PM; medical camp began around 2:40 PM |
| Subject | Village/para-level model meeting and community orientation on the project. |
| Participants | Karbari, BRAC worker, adult residents, children, three engagement field assistants, engagement group leader Kraingkong Mro, and Medical Assistant Manjuk Chakma. Total mentioned: 42 people. |
| Venue | Kompong Para, Borkola Jhiri Zone, Lama, Bandarban |
| Reported by | Kraingkong Mro, Engagement Group Leader, Dev Care Foundation |

### Agenda / Topics Discussed

- Introductions and welcome remarks.
- Introduction of para worker, BRAC worker, MVDA staff, and their responsibilities.
- Flipchart presentation on Vaccine for All and Medicine for All.
- Collection of community opinions and community-level agreement or permission.
- Video screening, karbari's speech, closing remarks, lunch, and medical camp.

### Activities / Proceedings

Under the direction of Project Coordinator Dr. Rupam Tripura and with support from Engagement Coordinator Mr. Saleh Ahmed, the team traveled from Lama office by auto, boat, and on foot. They reached Kompong Para around midday after a 30–40-minute walk from the landing point. After greetings and introductions, the group leader presented the flipchart on Vaccine for All and Medicine for All. Residents gave community-level agreement or permission to join the research and to receive medicine. The karbari spoke to increase confidence and trust. Lunch was served around 2:00 PM and the medical camp began around 2:40 PM.

### Questions and Responses

| **Question / Concern** | **Response / Explanation** |
| --- | --- |
| Why is the vaccine not being given in our village? | The document records this question but does not provide a specific written answer in the narrative section. |
| Why will pregnant women not receive medicine? | The question was raised by residents; detailed response is not written in the narrative section. |
| What happens if a pregnant woman accidentally takes the medicine? | A related brief record says the pregnant mother would be observed until delivery and after delivery, with regular contact by the para health worker. |
| Community suggestions | More meetings should be arranged; meetings are better at night around 6:00-7:00 PM; video should be shown on a large screen because daytime conditions and limited space made viewing difficult. |

### Challenges / Observations

- Few people are available in the village during work hours.
- Communication/network problems.
- Transportation difficulties; some villages are risky to visit alone.

## 8. Chhalom Para - Current Ongoing Service

### Meeting Details

| **Village/Area** | **Chhalom Para, Borkola Jhiri Zone, Rupsi Para, Lama, Bandarban** |
| --- | --- |
| Date and Time | 08 October 2024; meeting opened 12:30 PM and ended 3:00 PM |
| Group | Current ongoing service |
| Participants | Chhalom Para residents, MVDA para health worker, engagement field assistants, engagement group leader, medical assistant, and local dignitaries. 50 participants (female 30, male 20); total present with dignitaries: 55. The para had 30 households and a total population of 203. |
| Venue | House of Chongnak Mro in Chhalom Para; the house is also used for other social meetings. |
| Purpose | Para-level model village meeting under the current ongoing service group. |
| Reported by | Mrs. Lipi Tripura, Engagement Group Leader, Dev Care Foundation, Lama, Bandarban |

### Agenda / Topics Discussed

- Welcome speech and introduction.
- Introduction of Dev Care Foundation as a non-profit organization working with the Government of Bangladesh on malaria elimination in Lama and Alikadam Upazilas.
- Presentation on project activities and the Current Ongoing Service group.
- Discussion of challenges/barriers and miscellaneous issues.
- Explanation in local language through poster and flipchart.

### Activities / Proceedings

The team explained that the Government of Bangladesh aims to eliminate malaria by 2030. The research is divided into four groups, one of which is Current Ongoing Service. Existing services such as mosquito-net use, keeping the surrounding environment clean, and rapid blood testing for malaria diagnosis should be strengthened. The team also explained that six drops of blood would be collected as DBS every six months, five times, and that community-level agreement or permission is required. The meeting ended with thanks and a request to the karbari to close the session.

### Questions and Responses

| **Question / Concern** | **Response / Explanation** |
| --- | --- |
| Is malaria elimination possible through current ongoing services? | The research has four groups. Current ongoing service includes strengthened net use, environmental cleanliness, and rapid blood testing. The research will compare which services are most effective; effective services may later be expanded to all. |
| Will the six drops of blood taken every six months be drawn from a vein like in hospital tests? | No. Six drops will be collected from a finger of the left hand. |
| Will blood from 50 people be collected only from our para? | No. It may be collected from about one or two people from each of 25 paras. |
| Can we get medicines for body pain, itching, or similar symptoms? | Medical assistants and nurses will assess patients and provide medicine based on their physical condition. |

### Challenges / Observations

- Jhum work season makes it hard to gather everyone.
- Transportation is difficult; after vehicle or boat travel, 45-60 minutes of walking may be required.
- Clean water and sanitation challenges in the field.
- Remote paras create network problems and slow information exchange.

## 9. Chingkum Mro Para - Brief Village-ID Record

### Meeting Details

| **Village ID** | **56** |
| --- | --- |
| Group | Vaccine for All |
| Zone/Para | Borkola Jhiri Zone; Chingkum Mro Para |
| Date | 08 October 2024, Tuesday |
| Venue | Kiang, Chingkum Mro Para, Lama, Bandarban |
| GPS | Collected for the para |
| Chairperson | Para karbari |
| Facilitation/Presentation | Facilitated by Pran Pao Mro, MVDA para health worker; MVDA activities presented by Monika Khiang, engagement field assistant. |
| Attendance | 76 participants; male 29, female 47 |
| Participants | Bhante, karbari, teacher, Ekta project manager, students, BRAC para worker, community residents, and Dev Care staff. |
| Reported by | Moni Mukul Chakma, Engagement Group Leader, Dev Care Foundation |

### Activities / Proceedings

This brief record repeats some Chingkum Mro Para information in a compact village-ID format. It is included once here for completeness.

### Questions and Responses

| **Question / Concern** | **Response / Explanation** |
| --- | --- |
| Can the vaccine be provided in our para for all residents? | Yes. The vaccine can be provided in your para. |
| How should we contact you if physical problems occur after receiving the vaccine? | Residents should contact the para health worker in the para. A research physician is also available, and the team will keep continuous contact with the community. |

## 10. Chot0kola Jhiri - Brief Village-ID 052

### Meeting Details

| **Village ID** | **52** |
| --- | --- |
| Group | Vaccine and Medicine for All |
| Zone/Para | Borkola Jhiri Zone; Chhotkola Jhiri |
| Date | 08 October 2024, Tuesday |
| Venue | Kiang, Chingkum Mro Para, Lama, Bandarban |
| GPS | Collected for the para |
| Chairperson | Para karbari |
| Facilitation/Presentation | Facilitated by MVDA para health worker Mosammat Ismat Ara; MVDA activities presented by Tongleng Mro, engagement field assistant. |
| Attendance | 48 participants; male 23, female 25 |
| Participants | Karbari, imam, teacher, students, BRAC para worker, community residents, and Dev Care staff. |
| Reported by | Chathowai Marma, Engagement Group Leader, Dev Care Foundation |

### Activities / Proceedings

This short record preserves the key village-ID meeting information from the source document.

### Questions and Responses

| **Question / Concern** | **Response / Explanation** |
| --- | --- |
| After receiving the coronavirus vaccine, I had itching. Will that happen after this vaccine? | Such a problem is unlikely after this vaccine. If anything like that occurs, contact the para worker and the team will arrange necessary treatment. |
| Will there be any problem if all vaccine doses are not taken? | There will be no immediate problem, but taking all doses may help prevent malaria in the future. |

## 11. Kompong Para - Brief Village-ID Record

### Meeting Details

| **Village ID** | **53** |
| --- | --- |
| Group | Medicine for All |
| Zone/Para | Borkola Jhiri Zone; Kompong Para |
| Date | 08 October 2024, Tuesday |
| Venue | Karbari's courtyard, Lama, Bandarban |
| GPS | Collected for the para |
| Chairperson | Para karbari |
| Facilitation/Presentation | Facilitated by Singlok Mro, MVDA para health worker; MVDA activities presented by Jaj Tripura, engagement field assistant. |
| Attendance | 27 participants reported; male 19, female 9 |
| Participants | Karbari, BRAC health worker, residents, and Dev Care staff. |
| Reported by | Kraikshong Mro, Engagement Group Leader, Dev Care Foundation |

### Activities / Proceedings

This brief record overlaps with the longer Kompong Para narrative but is retained to preserve the village-ID data and specific Q&A.

### Questions and Responses

| **Question / Concern** | **Response / Explanation** |
| --- | --- |
| If a pregnant mother accidentally takes the medicine, what will you do? Will anything happen? | The pregnant mother will be kept under observation until delivery and after delivery. The para health worker will maintain continuous contact with her. |
| Why will the vaccine not be given in our para? | The paras were selected through a lottery. After checking vaccine effectiveness, the vaccine may also be provided in your para later. |

## 12. Chhalom Para - Brief Village-ID 055

### Meeting Details

| **Village ID** | **55** |
| --- | --- |
| Group | Current ongoing service |
| Zone/Para | Borkola Jhiri Zone; Chhalom Para |
| Date | 08 October 2024, Tuesday |
| Venue | Karbari's courtyard, Lama, Bandarban |
| GPS | Collected for the para |
| Chairperson | Menshing Mro, karbari |
| Facilitation/Presentation | Facilitated by Menpong Mro, MVDA para health worker; MVDA activities presented by Tomgpong Mro, engagement field assistant. |
| Attendance | 50 participants; male 30, female 20 |
| Participants | Member, karbari, teacher, students, BRAC para worker, community residents, and Dev Care staff. |
| Reported by | Lipi Tripura, Engagement Group Leader, Dev Care Foundation |

### Activities / Proceedings

This brief record is similar to the detailed Chhalom Para record and is included once to preserve the concise village-ID version.

### Questions and Responses

| **Question / Concern** | **Response / Explanation** |
| --- | --- |
| You said six drops of blood will be taken every six months, five times. Will this be taken from a vein like hospital blood collection? | No. Six drops of blood will be collected from a finger of the left hand. |

# 5. Consolidated Community Questions and Responses

| **Theme** | **Consolidated English Response** |
| --- | --- |
| Treatment costs after participation | If a health problem appears after research participation, the team will arrange continuous follow-up and treatment. Dev Care Foundation will bear necessary treatment costs. |
| Six-monthly blood collection / DBS | Blood will be collected to test for malaria parasites using advanced laboratory equipment. Several sections state that six drops of blood will be taken from a finger, not from a vein. |
| Where medicine or vaccine will be provided | Different paras have different arrangements. Janali Para residents were told medicine would be provided at Janali Para Government Primary School. Some Chingkum Mro Para residents were told the vaccine could be given in the para. Bidyamoni residents were told the initial sub-zone was Krongleng Para School. |
| Vaccine for neighboring villages | Selection was based on malaria case burden, local NGO information, lottery/group assignment, and the study design. Some communities were told that effective interventions may be provided later after results are assessed. |
| Side effects and health contact | Residents were advised to contact the para health worker or the project team if side effects or physical problems occur. Research physicians, medical assistants, and nurses were mentioned as available support. |
| Pregnant women and breastfeeding mothers | Pregnant women, breastfeeding mothers, and children below six months are excluded from the vaccine group in the source text. If a pregnant woman accidentally takes medicine, the source states that she will be kept under observation and the para health worker will maintain contact. |
| Allergy or chronic illness | If immediate treatment is required, vaccination will not be given at that time. People with long-term allergy or other chronic illness may be vaccinated if otherwise suitable. |
| Children | Children above six months may be eligible if they meet other criteria; children below six months are excluded. |
| Incomplete vaccine doses | One brief record state that there will be no immediate problem if all doses are not taken, but taking all doses may help prevent malaria in the future. |
| Community suggestions | Communities requested more meetings, evening meeting times, better/larger video display, and easier service locations where sub-zones were far from the para. Also it will very helpful if you can organize medical camp on regular basis. |

N.B. Based on the Model village meeting, we had conducted another 88 same types of village meeting.

# Part 2: Meetings with Karbaris

Meeting with Karbaris

Dev Care Foundation / MVDA Malaria Elimination Research

## Meeting Register

| **No.** | **Venue / Area** | **Date and Time** | **Participants** | **Main Focus** | **Reported by** |
| --- | --- | --- | --- | --- | --- |
| 1 | Boro Kolar Jhiri Primary School | 02 Jul 2024; 10:00 AM-3:00 PM | 29 total; 20 Karbaris + project staff | Project awareness with Karbaris; roles and health worker recruitment | Kraingkong Mro |
| 2 | Kurukpata Maitri Government Primary School | 02 Jul 2024; 10:00 AM-2:30 PM; EFA meeting 3:30-5:00 PM | 48 total; chairman, 34 Karbaris, 10 EFAs, MA, study nurse, group leader | Selected-para Karbari awareness and linkage meeting | Chathowai Marma (Hero) |
| 3 | Julaing Government Primary School | 02 Jul 2024; 10:30 AM-2:00 PM | 26 total; 18 Karbaris + project staff | Project activity discussion with EFAs and Karbaris | Mrs. Lipi Tripura |
| 4 | Tau Para Government Primary School | 02 Jul 2024; 11:30 AM-2:30 PM | 14 total; field staff, medical assistant, lab technologist, Karbaris | Project awareness with EFAs and Karbaris | Moni Mukul Chakma |
| 5 | Langri Para Government Primary School Hall / Tinku Para area | 02 Jul 2024; 10:00 AM-2:00 PM | 13 names listed; source says 7 Karbaris + 5 field assistants | MVDA project orientation, Karbari coordination, para worker recruitment | Pragati Chakma |

## Common Agenda Across the Meetings

- Welcome, greetings, and introduction of Dev Care Foundation staff, field assistants, Karbaris, chairman, medical staff, study nurse, and other participants.
- Project orientation on malaria elimination research and the MVDA project.
- Presentation of project activities in Mro and local/regional languages using leaflets and discussion.
- Explanation of the four study/service groups: vaccine and medicine with ongoing service; vaccine with ongoing service; medicine with ongoing service; and ongoing service only.
- Discussion of the role of Karbaris in community mobilization, rumor management, venue selection, meeting support, and encouraging participation.
- Discussion of MVDA para health worker recruitment, recruitment committees, application deadlines, and interview/test venues.
- Open discussion and question-and-answer session.
- Next-week work plan, thanks, and closing.

## Key Decisions and Follow-up Actions

- Karbaris were requested to cooperate with the project and support para-level implementation.
- Each selected para would have one para health worker recruited through a committee process involving Karbaris, Dev Care Foundation, and local NGO representatives where applicable.
- Eligible candidates for para health worker positions were asked to submit applications/CVs by 05 July 2024.
- Karbaris were requested to explain the positive aspects of the project to residents and help reduce rumors or misunderstandings.
- Karbaris and field assistants were asked to remain in regular contact in their assigned working areas.
- The project team emphasized fever testing, use of insecticide-treated bed nets, and maintaining clean surroundings as part of malaria prevention.

# Detailed Structured Meeting Reports

## 1. Meeting with Karbaris at Boro Kolar Jhiri Primary School

| **Date and Time** | **02 July 2024; meeting started at 10:00 AM, lunch break from 1:00-2:00 PM, and ended at about 3:00 PM.** |
| --- | --- |
| Venue | Boro Kolar Jhiri Primary School; held in the Grade 3 classroom. |
| Participants | 20 Karbaris, 1 Engagement Coordinator, 1 Engagement Group Leader, 1 Medical Assistant, and 6 Engagement Field Assistants; total 29 participants. |
| Purpose | To inform Karbaris at zone level about the project, project activities, their role in implementation, and field-level health worker recruitment. |
| Reported by | Kraingkong Mro, Engagement Group Leader, Dev Care Foundation, Lama, Bandarban Hill District. |

### Agenda / Topics Discussed

1. Detailed discussion about the project and its activities.

2. Presentation of project activities in Mro and regional languages.

3. Discussion of the Karbaris’ role in implementing the project.

4. Discussion of MVDA health worker recruitment requirements.

5. Discussion of the next week’s work plan.

6. Vote of thanks and closing.

### Activity Summary

The Engagement Coordinator and Engagement Group Leader first welcomed participants, introduced themselves, and explained the project. Field assistants then presented project activities in Mro and regional languages using leaflets. The meeting discussed how Karbaris would support implementation and work with para health workers. Recruitment notices were distributed, candidates were asked to submit CVs by 05 July 2024, and a recruitment committee was formed through discussion with the Karbaris. Recruitment activities were scheduled for 08 July 2024 at Boro Kolar Jhiri Primary School and 10 July 2024 at Naikhyangmukh Primary School. The meeting ran from 10:00 AM to around 3:00 PM, and video and photographs were preserved.

### Questions and Answers

| **Question / Concern** | **Response / Explanation** |
| --- | --- |
| Why will all villages not receive the vaccine? | The facilitators explained that 25 villages would first be selected by lottery. Depending on the quality/effectiveness of the medicine and vaccine, it may later be provided gradually to all villages. |
| After the COVID-19 vaccine, many people had problems. Will the same happen with this vaccine? | The facilitators explained that no major side effects were expected. Like other vaccines, swelling, redness, pain, or rare drowsiness may occur; participants were advised not to worry and to drink enough water. |
| When will vaccine and medicine distribution start? | The plan stated in the meeting was to begin around the beginning of the following year. |
| A previous NGO gave three doses of malaria medicine and did not follow up. Will that happen here? | The team stated that all first, second, and third doses would be administered directly in person, participants would be observed for 30 minutes, and field workers would remain in contact. |
| Some people previously became more affected by malaria after taking medicine. What guarantee is there for this medicine and vaccine? | The facilitators said this medicine is different from other malaria medicines and described the medicine and vaccine as supported by Oxford University and WHO prequalification, according to the source meeting record. |
| Will you support us if side effects occur? | The medical team would provide health support and regular follow-up if any issue occurs after medicine or vaccination. |
| Will elderly people also receive the vaccine? | Everyone above 6 months of age may receive the vaccine, except people with severe illness, pregnant women, breastfeeding mothers, and people staying outside their own area. |

## 2. Meeting with Selected-Para Karbaris at Kurukpata Maitri Government Primary School

| **Date and Time** | **02 July 2024; 10:00 AM-2:30 PM. There was a 30-minute break at 12:00 PM, followed by a meeting with Engagement Field Assistants from 3:30-5:00 PM.** |
| --- | --- |
| Venue | Kurukpata Maitri Government Primary School, located beside Kurukpata Bazar. |
| Participants | Total 48 participants: 1 chairman, 34 Karbaris, 10 Engagement Field Assistants, 1 Medical Assistant, 1 Study Nurse, and 1 Engagement Group Leader. |
| Purpose | To orient selected para Karbaris about the project, connect them with office staff and field assistants, explain their future support role, and support MVDA health worker recruitment. |
| Reported by | Chathowai Marma (Hero), Engagement Group Leader, MVDA Lama, Bandarban. |

### Agenda / Topics Discussed

1. Welcome speech and greetings.

2. Introduction session.

3. Presentation on project activities.

4. Discussion on how Karbaris will support project implementation.

5. Planning for the following week.

6. Thanks and closing.

### Activity Summary

The Engagement Group Leader welcomed the participants and introduced the organization. The Chairman of Kurukpata Union also delivered welcome remarks and guidance. Participants introduced themselves with their names, roles, and village names. Project activities were presented in Mro by Menpai Mro with support from Papai Mro, and in Tripura by Anaram Tripura with support from Ram Bahadur Tripura. The meeting discussed Karbaris’ responsibilities, including participation in recruitment committees, explaining benefits to residents, supporting para-level meetings, helping select venues, encouraging people to take vaccine and medicine, and reporting any rumors. A three-person recruitment committee model was discussed, involving a Karbari, Dev Care Foundation, and NGO representative. Recruitment was planned for 08 July 2024 at Kurukpata School and 10 July 2024 at Janali Para, with 36 health workers to be recruited.

### Questions and Answers

| **Question / Concern** | **Response / Explanation** |
| --- | --- |
| What is the purpose of the project? | To support malaria elimination by 2030 through work on “Vaccine for All” and “Medicine for All.” |
| How will the work be conducted? | The work will be conducted in lottery-selected paras under four groups: vaccine and medicine with ongoing service; vaccine with ongoing service; medicine with ongoing service; and ongoing service only. |
| Why are all paras not included? | The project focuses on paras where malaria cases were higher in the previous year, especially places where around 5 out of 100 people were found to have malaria. |
| On the recruitment day, can new Karbaris attend besides those present today? | The facilitators said only Karbaris from selected paras would attend, and the selected paras were announced in front of everyone. |
| Can the sub-zone location be changed from Krongley Para Government Primary School to Arui Para Missionary Centre? | The issue would be submitted to the authority; a decision could be communicated after the authority responds. |
| Is there an opportunity to submit new documents for health worker recruitment? | Yes. Documents could be submitted until 05 July 2024. |
| When will activities start? | After completing necessary steps, especially health worker recruitment, activities will start through para-level sessions. |
| Who will benefit from the project? | Residents over 6 months of age who live permanently in the para and provide consent will be eligible for project benefits. |

## 3. Meeting with Karbaris at Lulaing Government Primary School

| **Date and Time** | **02 July 2024; started at 10:30 AM and ended at 2:00 PM.** |
| --- | --- |
| Venue | Julaing Government Primary School, Sarai, Lama, Bandarban Hill District. The meeting was held in a quiet meeting room near a BRAC branch office, a Kyang, and a small bazar. |
| Participants | Field Assistants, Engagement Group Leader, Medical Assistant, and Karbaris. The source states that 18 Karbaris and 26 participants in total were present. |
| Purpose | To hold a project awareness meeting with Engagement Field Assistants and Karbaris on Dev Care Foundation project activities. |
| Reported by | Mrs. Lipi Tripura, Engagement Group Leader, Dev Care Foundation, Lama, Bandarban Hill District. |

### Agenda / Topics Discussed

1. Welcome speech.

2. Introduction session.

3. Presentation on project activities.

4. Discussion of the following week’s work plan.

5. Final remarks and closing.

### Activity Summary

The meeting began with introduction and welcome remarks by Mrs. Lipi Tripura. All participants introduced themselves. Project activities were presented in Mro by Menching Mro and in a local language by Bishwajit Tripura. The following week’s work plan was discussed in detail. The meeting also discussed MVDA health worker recruitment, committee formation, recruitment examination venues and times, and requested Karbaris to discuss the positive aspects of the project with community residents and attend para-level meetings.

### Questions and Answers

| **Question / Concern** | **Response / Explanation** |
| --- | --- |
| If we get malaria, will we always be able to get medicine or testing? | The team explained that medicine or vaccine would be provided before malaria occurs. If malaria occurs, residents should contact BRAC or Ekta health workers. |
| How often will we receive the vaccine? | Participants would receive a total of three doses at one-month intervals, followed by a booster dose after one year. |
| What should someone do if they cannot attend on the vaccine day? | They should contact the para health worker. If the health worker informs the project team, arrangements will be made. |
| Will the medicine always be available? | No. It would be provided once per month for three consecutive days. |
| What will happen if pregnant women and children under 6 months do not receive vaccine or medicine? | The source document lists this question, but the answer is not recorded. |
| What should we do if someone suddenly gets fever? | They should contact BRAC or Ekta health workers and complete an RDT test. |

## 4. Meeting with Karbaris at Tao Para Government Primary School

| **Date and Time** | **02 July 2024; 11:30 AM-2:30 PM.** |
| --- | --- |
| Venue | Tau Para Government Primary School, Lama; held in a quiet meeting room. A small shop is located beside the school. |
| Participants | Field Assistants, Engagement Group Leader, Medical Assistant, Lab Technologist, and Karbaris; total 14 participants listed. |
| Purpose | To organize a Dev Care Foundation project awareness meeting with Karbaris and Engagement Field Assistants. |
| Reported by | Moni Mukul Chakma, Senior Engagement Group Leader, Dev Care Foundation, Bandarban/Lama. |

### Agenda / Topics Discussed

1. Welcome speech.

2. Introduction session.

3. Discussion about the project.

4. Planning for the following week.

### Activity Summary

Moni Mukul Chakma opened the meeting at 11:30 AM with welcome remarks. Participants introduced themselves. Groinam Mro presented the project activities in Mro, and Ringthara Tripura presented in the local language. Field Assistants used leaflets to explain the project. The meeting then discussed MVDA health worker recruitment, committee formation, selection of examination location and time, and requested Karbaris to communicate the project’s positive points to para residents. The meeting ended at 2:30 PM.

### Questions and Answers

| **Question / Concern** | **Response / Explanation** |
| --- | --- |
| If someone does not want to take the vaccine in the first phase, can they receive it later? | Yes. According to the research team’s decision, a later time may be fixed for vaccination. |
| Can someone take other medicine while receiving the vaccine? | Yes, but only according to the advice of research workers or health workers. |
| What problem may occur if vaccine or medicine is given to children under 6 months? | The source document lists this question, but the answer is not recorded. |
| Many people had problems after the COVID-19 vaccine. Will this vaccine cause problems? | The facilitators explained that the the vaccine had undergone safety assessment; according to the source, it was developed by Oxford University, produced by the Serum Institute of India, and has 77% efficacy. |
| Why were only 100 paras selected when there are many paras? | The team explained that, among 16,567 malaria patients in Bangladesh in 2023, 10,001 were in Lama and Alikadam. These 100 paras had a higher malaria burden and were therefore selected. |
| Why will pregnant women not receive vaccine or medicine? | The source document lists this question, but the answer is not recorded. |

## 5. Meeting at Langri Para Government Primary School Hall / Tinku Para Area

| **Date and Time** | **02 July 2024, Tuesday; 10:00 AM-2:00 PM. Registration began from 9:30 AM.** |
| --- | --- |
| Venue | Langri Para Government Primary School hall room, on the road from Alikadam to Thanchi; the nearby Langri Para is included in the project. |
| Participants | Selected MVDA para Karbaris and field assistants. The source states 7 Karbaris and 5 field assistants, while 13 names are listed. |
| Purpose | To inform Karbaris about the MVDA project, involve them in the project, and request their cooperation for project work. |
| Reported by | Pragati Chakma, Lab Technologist, MVDA Project, Lama Office, Bandarban. |

### Agenda / Topics Discussed

1. Welcome remarks and introduction.

2. Discussion on the MVDA project.

3. Coordination between Karbaris and field assistants.

4. Para worker recruitment and next week’s work plan.

5. Open discussion.

6. Current malaria situation.

7. Miscellaneous items and closing.

### Activity Summary

Registration took place from 9:30-10:00 AM. Lab Technologist Pragati Chakma welcomed participants and gave a general introduction to MVDA and malaria. Field Assistant Ruai Mro presented the project in the Mro language, explaining Bangladesh’s target of eliminating malaria by 2030, the selection of 100 malaria-prone paras in Lama and Alikadam, and the division of paras into four groups of 25: vaccine and medicine, medicine only, vaccine only, and current ongoing service. Participants were told that a village may be selected by lottery into any group. Karbaris were described as a key driving force for project implementation, and field assistants were asked to maintain contact with the Karbaris in their areas. The meeting also covered recruitment of one para worker per selected para, pending applications, and the need for Karbaris to attend recruitment. Karbaris were asked to discuss the current malaria situation in their paras. Participants were reminded that malaria is more common in the rainy season, advised to use insecticide-treated bed nets, and encouraged to get free malaria testing immediately if fever occurs.

### Questions and Answers

| **Question / Concern** | **Response / Explanation** |
| --- | --- |
| If 25 paras receive vaccine and medicine while other paras receive only vaccine or current ongoing service, will people from other paras feel upset? | The team explained that the project has four groups and the approach that benefits people most may be applied later. It should be explained that malaria elimination is possible in any MVDA project group. |
| Will the vaccine cause dizziness? | The facilitator said the vaccine should not cause dizziness. As with other vaccines, there may be mild pain at the injection site or slight fever, but there is no reason to fear. The MVDA medical team will be available for any health issue. |
| If malaria is not eliminated through current ongoing service only, will vaccine or medicine later be provided to that village? | The facilitator explained that malaria can be eliminated through current ongoing services if people sleep under insecticide-treated nets, keep surroundings clean, and remain aware of mosquito bites. The MVDA team will follow up; if elimination is not achieved later, vaccine or medicine may be provided. |
| Should we take malaria treatment from Dev Care Foundation or from BRAC? | Malaria treatment should be taken from BRAC. If residents contact Dev Care Foundation staff, the staff will help connect them with BRAC workers. |

# Consolidated Themes from Community Questions

| **Theme** | **Community Concern** | **Typical Response Given** |
| --- | --- | --- |
| Selection of paras | Why are some paras selected while others are not? | Selected paras were chosen based on malaria burden, lottery allocation, and study grouping. Facilitators explained that effective approaches may later be expanded. |
| Safety and side effects | Will the vaccine or medicine cause problems like people experienced after COVID-19 vaccination? | Facilitators said serious problems were not expected; minor pain, redness, swelling, fever, or drowsiness may occur. The medical team would follow up and provide support. |
| Eligibility | Who can receive vaccine/medicine? | People above 6 months of age who live in the selected area and consent may participate, except those with severe illness, pregnant women, breastfeeding mothers, and people staying outside their own area. |
| Doses and schedule | How often will vaccine/medicine be given? | The vaccine was described as three doses at one-month intervals, with a booster after one year. Medicine availability was described as once per month for three consecutive days. |
| Follow-up care | What happens if someone becomes ill or has side effects? | Residents were told to contact para health workers, project medical staff, or BRAC/Ekta health workers; follow-up and health support would be arranged. |
| Treatment pathway | Should malaria treatment be taken from Dev Care Foundation or BRAC? | Treatment should be taken through BRAC; Dev Care staff could help connect residents with BRAC workers. |

# Part 3: Special Meetings Before Intervention

Special Meeting Before Intervention

# Consolidated Meeting Register

1. Janali Bazar Para / Jira Para - 4 November 2024. Source gives one time as 6:30 p.m. and another as 11:00 a.m. to 1:00 p.m.. Attendance: 7 participants: Janali Bazar Para karbari, 2 field assistants, 1 group leader, 1 engagement coordinator, and the person proposed for recruitment. Main outcome: Shahina Akter was finalized for recruitment as the MVDA village health worker for Janali Bazar Para.

2. Ukkling Para - 6 November 2024, 4:00 p.m. to 6:20 p.m.. Attendance: 39 participants: 21 women and 18 men. Participants included the village karbari Ukkling Mro, Mro commander Mendon Mro, ward member Suisang Mro, Langring Para karbari Tulo Mro, health inspector Mrinal Kanti Barua, health assistant Liton Kanti Shil, Ekota program organizer Sheikh Ahmed, and community members. Main outcome: Villagers and the karbari gave verbal agreement to accept vaccination on condition that key local leaders first receive the vaccine in front of them and that they can observe results in other villages.

3. Langring Para - 12 November 2024, 10:00 a.m. to 12:30 p.m.. Attendance: 30 participants: 15 women and 15 men. Participants included the karbari, Mro commander, ward member, health inspector, health assistant, group leader, field assistant, village health worker, engagement coordinator, and community members. Main outcome: Participants agreed to take part in the research after explanation. The report recommends more coordination with the health department and courtyard meetings to address broader vaccination awareness.

4. Talai Para - 14 November 2024, 10:00 a.m. to 1:00 p.m.. Attendance: 23 participants: 12 women and 11 men. Participants included the karbari, MVDA village health worker, BRAC para worker, and community members. Main outcome: Villagers were informed of the change and reassured that they may be considered in a later phase if possible.

5. Sitajhiri Para - 14 November 2024, 12:00 p.m. to 1:00 p.m.. Attendance: 15 participants: 8 women and 7 men. Participants included the karbari, MVDA village health worker, BRAC para worker, and community members. Main outcome: Villagers were informed and reassured about possible future inclusion.

6. Tiarjhiri Para - 14 November 2024, 12:00 p.m. to 3:00 p.m.. Attendance: 41 participants: 15 women and 26 men. Participants included the karbari, MVDA village health worker, BRAC para worker, and community members. Main outcome: All present promised to participate in the research and sign the consent form.

7. Luleng Bazar Para - 15 November 2024, 10:20 a.m. to 12:30 p.m.. Attendance: 23 participants: 10 women and 13 men. Participants included the karbari, MVDA village health worker, BRAC para worker, and community members. Main outcome: Participants assured the team that they would participate in the research.

8. Manglai Para - 2 December 2024, 11:30 a.m. to 12:30 p.m.. Attendance: 23 participants, including the karbari, MVDA village health worker, BRAC para worker, group leader, engagement field assistant, and villagers. Main outcome: Lemu Palang Government Primary School was preferred by the community as the more convenient intervention site.

9. Thanda Jhiri Para - 4 December 2024, 11:35 a.m. to 12:30 p.m.. Attendance: 11 participants, including two engagement group leaders, one family planning assistant, one engagement field assistant, one village health worker, the karbari, and community members. Main outcome: Families received additional explanation, but some concerns about children remained and were to be discussed further by the families.

10. Hamuk Para - 15 December 2024, 12:00 p.m. to 2:30 p.m.. Attendance: 15 participants, including the karbari, community members, village health worker, group leader, and engagement field assistant. Main outcome: Many villagers agreed to provide written consent after the explanation.

11. Mengta/Reang Para - 11 December 2024, 10:00 a.m. to 2:00 p.m.. Attendance: 14 participants, including research physician, medical assistant, karbari, community members, village health worker, group leader, and field assistant. Main outcome: Participants understood the clarification and agreed to join the research and accept vaccine and medicine.

12. Ramju Para - 11 December 2024, 12:20 p.m. to 1:30 p.m.. Attendance: 11 participants, including karbari, community members, village health worker, group leader, field assistant, and BRAC health worker. Main outcome: Consent was collected from the two families, but a follow-up community decision about small children was still expected.

13. Langchang Para - 8 December 2024, 11:30 a.m. to 1:00 p.m.. Attendance: 15 participants, including karbari, villagers, village health worker, group leader, field assistant, and BRAC health worker. Main outcome: Villagers received clarification on consent, intervention site, and support arrangements.

14. Tiarjhiri Marma Para - Source is inconsistent: date line says 15 December 2024 at 11:00 a.m.; another line says 8 December 2024, 11:00 a.m. to 1:00 p.m.. Attendance: 20 participants, including karbari, villagers, village health worker, group leader, and engagement field assistant. Main outcome: Participants agreed to consent after receiving information in Marma language. A health camp was also held.

15. Choto Kalar Jhiri Para - 3 January 2025, 11:00 a.m. to 12:30 p.m.. Attendance: 17 participants, including karbari, villagers, village health worker, group leader, field assistant, engagement coordinator, and mosque imam. Main outcome: The hesitant families’ doubts were resolved and consent was collected.

16. Lakhan Jhiri Bangali Para - 3 January 2025, 11:50 a.m. to 12:45 p.m.. Attendance: 19 participants, including karbari, villagers, village health worker, group leader, field assistant, and mosque imam. Main outcome: A Ramadan-friendly vaccination schedule was agreed in principle.

17. New Muslim Para - 3 February 2025, 11:00 a.m. to 1:30 p.m.. Attendance: 30 participants: 12 men and 18 women. Participants included karbari, villagers, village health worker, group leader, field assistant, mosque imam, and hostel teacher. Main outcome: The Ramadan vaccination plan was accepted, and the medical camp increased community satisfaction.

18. Janali Para - 3 February 2025, 3:30 p.m. to 5:00 p.m.. Attendance: 32 participants: 15 men and 17 women. Participants included karbari, MVDA village health worker, group leader, engagement field assistant, mosque imam, and villagers. Main outcome: The community agreed to a Ramadan-compatible medicine distribution arrangement.

19. Krangleng Para - 14 January 2025, 6:30 p.m. to 8:10 p.m.. Attendance: 32 participants, including the karbari, five monks/religious leaders, health assistant, Ekota program organizer, engagement coordinator, anthropologist, group leader, field assistant, and villagers. Main outcome: The meeting clarified the purpose of vaccine and medicine, but the source indicates deep-rooted vaccine hesitancy in the community.

20. Chiuni / Palir Jhiri Para - Source is inconsistent: one field says 4 February 2025 at 11:00 a.m.; another says 3 February 2025, 4:00 p.m. to 5:00 p.m.. Attendance: 25 participants: 10 men and 15 women. Participants included karbari, villagers, village health worker, group leader, engagement field assistant, and mosque imam. Main outcome: A Ramadan medicine intake plan was agreed, with assurance that allowances would still be provided.

# Detailed Structured Reports

## 1. Janali Bazar Para / Jira Para: Replacement of MVDA Village Health Worker

| **Date / Time** | **4 November 2024. Source gives one time as 6:30 p.m. and another as 11:00 a.m. to 1:00 p.m.** |
| --- | --- |
| Place | Rambahadur’s house, Jira Para. Village ID was not written in the source. |
| Participants / Attendance | 7 participants: Janali Bazar Para karbari, 2 field assistants, 1 group leader, 1 engagement coordinator, and the person proposed for recruitment. |
| Purpose | To address complaints from Janali Bazar Para that a person from another para had been recruited as the MVDA village health worker, and to decide on a locally acceptable worker. |
| Outcome / Decision | Shahina Akter was finalized for recruitment as the MVDA village health worker for Janali Bazar Para. |
| Reported by | Chathowai Marma, Engagement Group Leader. |

### Agenda / Discussion Points

- Discuss the recruitment of the MVDA village health worker.
- Consult the karbari and community members.
- Resolve community concerns before the intervention.

### Activities / Proceedings

- The team discussed the matter with the karbari and community representatives.
- The community explained that they preferred a Bengali worker from Janali Bazar Para so that local communication would be easier.
- The meeting decided to recruit Shahina Akter, a permanent resident of Janali Bazar Para, as the MVDA village health worker.

### Questions and Answers

No specific question-and-answer section was recorded in the source for this meeting.

### Source Notes

Personal review states that during the model meeting on 8 October 2024, villagers said they might not participate in the research if a worker from their own para was not appointed.

## 2. Ukkling Para: Special Trust-Building Meeting Before Vaccination

| **Date / Time** | **6 November 2024, 4:00 p.m. to 6:20 p.m.** |
| --- | --- |
| Place | Initially in the courtyard beside the karbari’s house; moved to a nearby house after sudden rain. |
| Participants / Attendance | 39 participants: 21 women and 18 men. Participants included the village karbari Ukkling Mro, Mro commander Mendon Mro, ward member Suisang Mro, Langring Para karbari Tulo Mro, health inspector Mrinal Kanti Barua, health assistant Liton Kanti Shil, Ekota program organizer Sheikh Ahmed, and community members. |
| Purpose | To rebuild confidence and support informed participation in the “Vaccine for All and Medicine for All” malaria research before intervention. |
| Outcome / Decision | Villagers and the karbari gave verbal agreement to accept vaccination on condition that key local leaders first receive the vaccine in front of them and that they can observe results in other villages. |
| Reported by | Saleh Ahmed, Engagement Coordinator; Fazle Rabbi, Anthropologist; Group Leaders Monimukul Chakma, Chatowai Marma, and Kraingkong Mro. |

### Agenda / Discussion Points

- Discuss the effectiveness and safety of the malaria vaccine.
- Clarify whether consent can be withdrawn after signing.
- Respond to community concerns that they wanted to see others vaccinated first.
- Discuss the community’s condition that the Mro commander and ward member should take the vaccine publicly before villagers do.

### Activities / Proceedings

- The team explained the vaccine using a flipchart poster.
- A project video was shown.
- The community asked questions about safety, consent, and whether the vaccine would also be provided in other villages.

### Questions and Answers

| **Question / Concern** | **Response / Clarification** |
| --- | --- |
| Will vaccination be provided only in our para, or in other paras too? | The research includes 100 paras: 56 in Lama Upazila and 44 in Alikadam Upazila. They are divided into four arms: vaccine plus medicine, vaccine only, medicine only, and existing service. |
| People say some become unconscious or die after vaccination. How effective and safe is this vaccine? | The team said the the vaccine had undergone safety assessment. Common mild effects may include redness, swelling or pain at the injection site, mild fever, drowsiness, or convulsions similar to other vaccines. |
| Why do you need a signature or thumbprint when other vaccines do not require it? | The consent form is a written record of willingness to participate. One copy stays with the participant and one copy is stored by the office. It also helps prevent later confusion or misunderstanding. |

### Source Notes

The personal review describes Ukkling Para as remote, about 6 km from Tinku Para Zone and around 40 minutes on foot from the main road through steep hills. The source notes long-standing mistrust of government and non-government organizations because of land-related concerns and fear of signing documents.

## 3. Langring Para: Follow-Up Meeting on Vaccine Fear and Acceptance

| **Date / Time** | **12 November 2024, 10:00 a.m. to 12:30 p.m.** |
| --- | --- |
| Place | Meeting house beside the karbari’s house, Langring Para, Village ID 100, Tinku Para Zone, Alikadam. |
| Participants / Attendance | 30 participants: 15 women and 15 men. Participants included the karbari, Mro commander, ward member, health inspector, health assistant, group leader, field assistant, village health worker, engagement coordinator, and community members. |
| Purpose | To address fear of vaccination and strengthen trust and acceptance so that the community could participate without anxiety. |
| Outcome / Decision | Participants agreed to take part in the research after explanation. The report recommends more coordination with the health department and courtyard meetings to address broader vaccination awareness. |
| Reported by | Saleh Ahmed, Engagement Coordinator; Fazle Rabbi, Anthropologist; Chatowai Marma, Engagement Group Leader. |

### Agenda / Discussion Points

- Registration.
- Welcome remarks and introductions.
- Presentation on MVDA research using flipchart/poster.
- Collection of participant opinions.
- Question and answer session.
- Chairperson’s remarks and closing.

### Activities / Proceedings

- Karbari and guests provided correct information on vaccination and explained the importance of the vaccine for malaria elimination.
- The team discussed that the para had not previously received EPI, COVID-19, or other vaccination services, which contributed to fear.
- The health inspector and health assistant noted that villagers usually did not attend vaccination centers.

### Questions and Answers

| **Question / Concern** | **Response / Clarification** |
| --- | --- |
| We have never taken vaccines before. Will vaccination be painful? | The the vaccine had undergone safety assessment. Mild injection-site pain, redness, swelling, mild fever, drowsiness, or convulsion may occur as with other vaccines. |
| Does everyone need to receive the vaccine? | Yes. Those living in the para, giving informed consent, and aged over 6 months will receive it. |
| How many paras in Alikadam will receive the vaccine? | The research includes 44 paras in Alikadam: 11 vaccine plus medicine, 11 vaccine only, 11 medicine only, and 11 continuing with existing service. |

### Source Notes

Personal review notes that many families had never vaccinated their children, and some children had no birth registration. The para was described as underserved and distant from public services.

## 4. Talai Para: Meeting to Explain Exclusion From the Research Area

| **Date / Time** | **14 November 2024, 10:00 a.m. to 1:00 p.m.** |
| --- | --- |
| Place | Karbari’s house, Talai Para. The para is about 7 km from Munai Bazar and around 50 minutes on foot; 23 families live there. |
| Participants / Attendance | 23 participants: 12 women and 11 men. Participants included the karbari, MVDA village health worker, BRAC para worker, and community members. |
| Purpose | To inform villagers that Talai Para had initially been selected as a research work area but was later dropped after the research arms were finalized by lottery. |
| Outcome / Decision | Villagers were informed of the change and reassured that they may be considered in a later phase if possible. |
| Reported by | Lipi Tripura, Group Leader, Luleng Zone. |

### Agenda / Discussion Points

- Explain the original selection of the para.
- Explain the later exclusion after lottery-based division into four arms.
- Prevent confusion among villagers who had been connected with the study for around nine months.

### Activities / Proceedings

- No games or special event could be organized.
- The team explained that the para is a malaria-prone area and may be considered later if an opportunity arises.

### Questions and Answers

| **Question / Concern** | **Response / Clarification** |
| --- | --- |
| Since our para has been dropped in the first phase, will we be able to receive the vaccine later? | If vaccination is provided in a later research stage, everyone in the para will be considered for vaccination. |

### Source Notes

The source states that the para had been linked with MVDA activities for around nine months before exclusion.

## 5. Sitajhiri Para: Meeting to Explain Exclusion From the Research Area

| **Date / Time** | **14 November 2024, 12:00 p.m. to 1:00 p.m.** |
| --- | --- |
| Place | House of the MVDA village health worker, Sitajhiri Para. The para is about 4 km from Rupsipara Bazar and around 50 minutes on foot; 6 families live there. |
| Participants / Attendance | 15 participants: 8 women and 7 men. Participants included the karbari, MVDA village health worker, BRAC para worker, and community members. |
| Purpose | To inform villagers that Sitajhiri Para was initially selected as a research area but was later excluded after the paras were divided into four arms by lottery. |
| Outcome / Decision | Villagers were informed and reassured about possible future inclusion. |
| Reported by | Kraingkong Mro, Group Leader, Luleng Zone. |

### Agenda / Discussion Points

- Explain why the para was excluded after initial selection.
- Clarify that the para had been connected with MVDA activities for around nine months.
- Reassure villagers and prevent misunderstanding.

### Activities / Proceedings

- No games or other event could be arranged.
- The team explained that the para is malaria-prone and may be included later if possible.

### Questions and Answers

| **Question / Concern** | **Response / Clarification** |
| --- | --- |
| Since our para has been excluded in the first phase, will we be able to receive the vaccine later? | If vaccine is provided later in the research period, everyone in the para will be considered for vaccination. |

### Source Notes

This report has almost the same structure and content as the Talai Para report.

## 6. Tiarjhiri Para: Participation and Written Consent Meeting

| **Date / Time** | **14 November 2024, 12:00 p.m. to 3:00 p.m.** |
| --- | --- |
| Place | Karbari’s house, Tiarjhiri Para. The para is about 5 km from Rupsipara Bazar and around 1 hour 20 minutes on foot. The venue was shaded and open enough for comfortable seating. |
| Participants / Attendance | 41 participants: 15 women and 26 men. Participants included the karbari, MVDA village health worker, BRAC para worker, and community members. |
| Purpose | To explain the benefits of participating in the research and whether all family members would sign the written consent form before joining. |
| Outcome / Decision | All present promised to participate in the research and sign the consent form. |
| Reported by | George Tripura, Engagement Field Assistant, Baraklajhiri Zone. |

### Agenda / Discussion Points

- Welcome remarks.
- Introduction session.
- Presentation of the research using a poster.
- Discussion on whether villagers would sign consent forms.
- Opinion collection and Q&A.
- Karbari’s remarks and closing.

### Activities / Proceedings

- Field Assistant George Tripura gave the welcome remarks with the karbari’s permission.
- Krajairy Marma presented MVDA activities using the poster.
- Villagers shared their opinions and questions after the presentation.
- No games or other special event could be organized.

### Questions and Answers

| **Question / Concern** | **Response / Clarification** |
| --- | --- |
| If malaria occurs after taking this medicine, can we take malaria medicine again? | Yes. If malaria occurs, malaria medicine can be taken again without a problem. |

### Source Notes

The meeting was mainly designed to secure family-level written consent before intervention.

## 7. Luleng Bazar Para: Follow-Up Meeting for Absentees and Consent Discussion

| **Date / Time** | **15 November 2024, 10:20 a.m. to 12:30 p.m.** |
| --- | --- |
| Place | BRAC branch office at Luleng Bazar Para. |
| Participants / Attendance | 23 participants: 10 women and 13 men. Participants included the karbari, MVDA village health worker, BRAC para worker, and community members. |
| Purpose | To meet people who were absent from an earlier meeting and explain participation benefits, written consent, and research requirements. |
| Outcome / Decision | Participants assured the team that they would participate in the research. |
| Reported by | Ushainui Marma, Engagement Field Assistant, Luleng Zone. |

### Agenda / Discussion Points

- Welcome remarks.
- Introductions.
- Research presentation using poster.
- Discussion on written consent.
- Opinion and Q&A session.
- Karbari’s remarks and closing.

### Activities / Proceedings

- Ushainui Marma greeted participants and conducted introductions.
- The team explained that Bangladesh aims to eliminate malaria by 2030.
- Participants were advised to keep surroundings clean, use mosquito nets before sleeping, test blood promptly during fever, and take medicine if malaria is confirmed.
- Research participants were told that DBS blood collection would be taken five times at six-month intervals, three drops each time.

### Questions and Answers

| **Question / Concern** | **Response / Clarification** |
| --- | --- |
| Can malaria be eliminated through existing services only? | The current existing service may not be sufficient by itself. The researchers hope that if vaccine and medicine are effective in intervention paras, they can later be provided in areas currently under existing service. |

### Source Notes

The same meeting record appears twice in the source document with identical details.

## 8. Manglai Para: Selection of a Convenient Intervention Site

| **Date / Time** | **2 December 2024, 11:30 a.m. to 12:30 p.m.** |
| --- | --- |
| Place | Karbari’s house, Manglai Para. The para is around 1 hour 30 minutes on foot from Moratto Bazar in Sarai Union. |
| Participants / Attendance | 23 participants, including the karbari, MVDA village health worker, BRAC para worker, group leader, engagement field assistant, and villagers. |
| Purpose | To discuss where Manglai Para residents should attend during the intervention so that walking time and access would be easier. |
| Outcome / Decision | Lemu Palang Government Primary School was preferred by the community as the more convenient intervention site. |
| Reported by | Lipi Tripura, Group Leader, Luleng Zone. |

### Agenda / Discussion Points

- Welcome remarks.
- Introductions.
- Discussion of main zone and sub-zone.
- Short discussion on the consent form.

### Activities / Proceedings

- The group leader Lipi Tripura greeted participants.
- The team explained that campaign activities in Luleng Zone would run at two locations: Lemu Palang Government Primary School and Luleng Government Primary School.
- It would take around 1 hour 30 minutes to reach Luleng Government Primary School, but around 50 minutes to reach Lemu Palang Government Primary School.
- Villagers preferred Lemu Palang Government Primary School and agreed to sign the consent form.

### Questions and Answers

| **Question / Concern** | **Response / Clarification** |
| --- | --- |
| Will the medicine distribution date be told in advance? | The village health worker and karbari will visit each household the day before medicine distribution so no family is missed. |
| Can medicine distribution be scheduled on Sunday market day, which is convenient for us? | The team will consult the authority and try. If not possible, villagers should attend on the assigned date. An honorarium/compensation for the missed workday will be provided. |

### Source Notes

No games, drama, or other special event could be arranged.

## 9. Thanda Jhiri Para: Re-Engagement With Families Reluctant to Take Vaccine and Medicine

| **Date / Time** | **4 December 2024, 11:35 a.m. to 12:30 p.m.** |
| --- | --- |
| Place | Karbari’s house, Thanda Jhiri Para. |
| Participants / Attendance | 11 participants, including two engagement group leaders, one family planning assistant, one engagement field assistant, one village health worker, the karbari, and community members. |
| Purpose | To revisit two of five families that were reluctant to take vaccine and medicine and explain the benefits again. |
| Outcome / Decision | Families received additional explanation, but some concerns about children remained and were to be discussed further by the families. |
| Reported by | Lipi Tripura, Engagement Group Leader, Dev Care Foundation, Lama, Bandarban. |

### Agenda / Discussion Points

- Welcome remarks and introductions.
- Discussion using leaflet and flipchart.
- Discussion on vaccine and medicine.
- Discussion on consent.
- Miscellaneous discussion.

### Activities / Proceedings

- Lipi Tripura opened the meeting and thanked the karbari and villagers.
- Leaflet and flipchart were used to explain the vaccine and medicine.
- Family Planning Assistant Somori Mro explained the importance of the vaccine and medicine in the local Mro language.
- The team learned that while adults in three families were willing, some families did not want young members to receive vaccine and medicine. They said they would discuss among themselves and inform the team the next day.

### Questions and Answers

| **Question / Concern** | **Response / Clarification** |
| --- | --- |
| Our grandparents did not take malaria vaccine or medicine. Why do we need it? | The response explained that circumstances have changed, people’s disease resistance is lower, and tested vaccines and medicines are safe. |

### Source Notes

The report emphasizes use of local Mro language to address reluctance.

## 10. Hamuk Para: Re-Meeting on Written Consent Concerns

| **Date / Time** | **15 December 2024, 12:00 p.m. to 2:30 p.m.** |
| --- | --- |
| Place | Courtyard of Karbari Kaipre’s house, Hamuk Para. |
| Participants / Attendance | 15 participants, including the karbari, community members, village health worker, group leader, and engagement field assistant. |
| Purpose | To address reluctance to sign written consent for the vaccine and medicine group. |
| Outcome / Decision | Many villagers agreed to provide written consent after the explanation. |
| Reported by | Kraingkong Mro, Group Leader, Barakla Jhiri Zone. |

### Agenda / Discussion Points

- Welcome remarks.
- Introduction session.
- Presentation using poster.
- Short discussion on consent form.

### Activities / Proceedings

- Group Leader Kraingkong Mro greeted participants and presented MVDA activities using the poster.
- The consent form was explained in detail, including the right to withdraw at any time.
- The contents of the consent form were explained in Mro language.
- Many participants understood the issues and gave consent during the meeting.

### Questions and Answers

| **Question / Concern** | **Response / Clarification** |
| --- | --- |
| After vaccinating dogs in our para, all dogs died. We are afraid something similar may happen with your vaccine. | The team said they did not know what vaccine had been given to dogs, but the MVDA the vaccine had undergone safety assessment. Mild fever may occur and should improve within one or two days with fluids. |
| Will you take responsibility if something happens? | The team will provide support for health needs and does not expect major problems because serious side effects are not expected. |

### Source Notes

No specific games, drama, or health education event could be arranged.

## 11. Mengta/Reang Para: Rumor Correction and Consent Meeting

| **Date / Time** | **11 December 2024, 10:00 a.m. to 2:00 p.m.** |
| --- | --- |
| Place | Courtyard of the karbari’s house, Mengta/Reang Para. |
| Participants / Attendance | 14 participants, including research physician, medical assistant, karbari, community members, village health worker, group leader, and field assistant. |
| Purpose | To address two families’ refusal to participate due to rumors that the vaccine or medicine could cause death. |
| Outcome / Decision | Participants understood the clarification and agreed to join the research and accept vaccine and medicine. |
| Reported by | Manjuk Chakma, Medical Assistant, MVDA, Lama. |

### Agenda / Discussion Points

- Welcome remarks.
- Introductions.
- Presentation using poster.
- Discussion on consent form.
- Discussion and correction of rumors.

### Activities / Proceedings

- Kraingkong Mro presented MVDA activities and explained the consent form.
- The team emphasized that consent can be withdrawn at any time.
- Rumors were discussed: that the medicine was expired and that the vaccine had never been used before.
- Dr. Ibrahim Khalil, Research Physician, clarified the issues in detail.
- The village health worker and karbari helped explain in Mro language.

### Questions and Answers

| **Question / Concern** | **Response / Clarification** |
| --- | --- |
| Will there be any problem if we take the vaccine? | The the vaccine had undergone safety assessment, with no serious side effects expected. It is 77% effective and has been used in Africa. Mild fever or body pain can occur with any vaccine and normally improves in one or two days. |

### Source Notes

The report highlights the role of local-language explanation by the village health worker and karbari.

## 12. Ramju Para: Follow-Up With Two Families Reluctant to Sign Consent

| **Date / Time** | **11 December 2024, 12:20 p.m. to 1:30 p.m.** |
| --- | --- |
| Place | Courtyard of the karbari’s house, Ramju Para. |
| Participants / Attendance | 11 participants, including karbari, community members, village health worker, group leader, field assistant, and BRAC health worker. |
| Purpose | To meet the karbari and two families who had refused written consent before vaccination, while other villagers had already consented. |
| Outcome / Decision | Consent was collected from the two families, but a follow-up community decision about small children was still expected. |
| Reported by | Lipi Tripura, Group Leader, MVDA, Lama. |

### Agenda / Discussion Points

- Welcome remarks.
- Introductions.
- Presentation using poster.
- Short discussion on consent form.

### Activities / Proceedings

- Lipi Tripura opened the meeting and presented MVDA activities.
- A BRAC para health worker was requested to explain in Mro language.
- The consent form was explained in detail, including the right to withdraw at any time.
- The two families were given the consent explanation in Mro and then consent was collected from them.
- Villagers still expressed fear about vaccinating small children; the karbari said they would hold another night meeting and inform the team about children.

### Questions and Answers

| **Question / Concern** | **Response / Clarification** |
| --- | --- |
| Will there be any problem if we take the vaccine? | The the vaccine had undergone safety assessment. It is 77% effective and has been used in Africa. Mild fever or body pain may occur, as with other vaccines, and should improve with fluids. The team will support health-service needs. |

### Source Notes

The report notes persistent fear around vaccinating children.

## 13. Langchang Para: Re-Meeting on Consent and Intervention Site

| **Date / Time** | **8 December 2024, 11:30 a.m. to 1:00 p.m.** |
| --- | --- |
| Place | Courtyard of the karbari’s house, Langchang Para. |
| Participants / Attendance | 15 participants, including karbari, villagers, village health worker, group leader, field assistant, and BRAC health worker. |
| Purpose | To hold another meeting because villagers were hesitant to provide consent in a vaccine-plus-medicine group para. |
| Outcome / Decision | Villagers received clarification on consent, intervention site, and support arrangements. |
| Reported by | Monimukul Chakma, Group Leader, MVDA, Lama. |

### Agenda / Discussion Points

- Welcome remarks.
- Introductions.
- Presentation using poster.
- Short discussion on consent form.

### Activities / Proceedings

- The vaccine and medicine were discussed again using a poster/flipchart.
- The need for written consent was explained.
- Kraingkong Mro explained the issues in Mro language, and the community understood the explanations.

### Questions and Answers

| **Question / Concern** | **Response / Clarification** |
| --- | --- |
| Where will our villagers receive the vaccine? | Gila Para is the sub-zone and is about 30 minutes on foot. People from four paras will receive vaccination there. |
| Can we take only the medicine without the vaccine? | No. This para is assigned to the vaccine and medicine group, so both will be provided. |
| If anything happens after vaccination, how will we contact you and will you support us? | An MVDA village health worker has been appointed in the para and will stay in contact. The project team will support any health-service needs. |

## 14. Tiarjhiri Marma Para: Consent Discussion and Health Camp

| **Date / Time** | **Source is inconsistent: date line says 15 December 2024 at 11:00 a.m.; another line says 8 December 2024, 11:00 a.m. to 1:00 p.m.** |
| --- | --- |
| Place | Cherang house in Tiarjhiri Marma Para, where other para meetings are also held. |
| Participants / Attendance | 20 participants, including karbari, villagers, village health worker, group leader, and engagement field assistant. |
| Purpose | To discuss consent because villagers in Tiarjhiri Marma Para were reluctant to give consent for research participation. |
| Outcome / Decision | Participants agreed to consent after receiving information in Marma language. A health camp was also held. |
| Reported by | Chatowai Marma, Group Leader, MVDA, Lama. |

### Agenda / Discussion Points

- Welcome remarks.
- Introductions.
- Presentation using poster.
- Short discussion on consent form.
- Karbari’s remarks.

### Activities / Proceedings

- The meeting opened with welcome remarks and introductions.
- Chatowai Marma, Group Leader, explained the research and consent form in Marma language.
- Participants understood the importance of the consent form and agreed to give consent.
- A health camp was conducted during the meeting.

### Questions and Answers

| **Question / Concern** | **Response / Clarification** |
| --- | --- |
| If we go to the Mangpu Para Government Primary School sub-zone, will we receive travel allowance? It costs at least Tk. 240 from our para. | Every person coming to the sub-zone will receive travel allowance. |
| If we have a problem after taking medicine, will you help with hospital, transport, or treatment costs? | Yes. The project worker in the para will inform the team, and treatment and actual transport cost will be arranged if hospital care is needed. |
| Will medicine also be provided for other illnesses such as body pain and itching? | Yes. If other medicines are needed, the team will provide them. A health camp can also be arranged if needed. |

## 15. Choto Kalar Jhiri Para: Follow-Up With Three Families Refusing Consent

| **Date / Time** | **3 January 2025, 11:00 a.m. to 12:30 p.m.** |
| --- | --- |
| Place | A shop in Choto Kalar Jhiri Para. The para is described as a Bengali para whose residents migrated from the Rangpur area; it is about one hour by boat from Rupsi Para. |
| Participants / Attendance | 17 participants, including karbari, villagers, village health worker, group leader, field assistant, engagement coordinator, and mosque imam. |
| Purpose | To explain the study again to three families who did not want to participate or sign the consent form. |
| Outcome / Decision | The hesitant families’ doubts were resolved and consent was collected. |
| Reported by | Kraingkong Mro, Group Leader, MVDA, Lama. |

### Agenda / Discussion Points

- Welcome remarks.
- Introductions.
- Presentation using poster.
- Short discussion on consent form.
- Karbari’s remarks.

### Activities / Proceedings

- Saleh Ahmed, Engagement Coordinator, opened the meeting.
- The project objectives and benefits were explained using poster/leaflet.
- Doubts among the shopkeeper and two families were resolved.
- Consent was taken during the meeting.
- A health camp was conducted by Medical Assistant Prokash Chakma.

### Questions and Answers

| **Question / Concern** | **Response / Clarification** |
| --- | --- |
| If we go to the Mangpu Para Government Primary School sub-zone, will travel allowance be given? | Yes. Each person coming to the sub-zone will receive travel allowance. |
| If a problem happens after taking medicine, will support be provided for hospital, transport, or treatment costs? | Yes. Inform the project worker in the para; necessary treatment and actual transport cost will be arranged if hospital care is needed. |
| Will medicine be provided for illnesses other than malaria, such as body pain and itching? | Yes. Necessary medicines will be provided. If the para needs a health camp, that can also be arranged. |

### Source Notes

The report highlights that the meeting was arranged in a local shop where villagers usually hold informal meetings.

## 16. Lakhan Jhiri Bangali Para: Ramadan Vaccination Schedule Discussion

| **Date / Time** | **3 January 2025, 11:50 a.m. to 12:45 p.m.** |
| --- | --- |
| Place | Karbari’s house, Lakhan Jhiri Bangali Para. The para is remote from Lama Sadar, surrounded by Mro settlements, and most residents cultivate tobacco and vegetables near a small hill stream. |
| Participants / Attendance | 19 participants, including karbari, villagers, village health worker, group leader, field assistant, and mosque imam. |
| Purpose | To discuss how vaccination should be conducted during Ramadan. |
| Outcome / Decision | A Ramadan-friendly vaccination schedule was agreed in principle. |
| Reported by | Monimukul Chakma, Group Leader, MVDA, Lama. |

### Agenda / Discussion Points

- Welcome remarks.
- Karbari’s remarks.

### Activities / Proceedings

- The community decided that small children and those not fasting would go to the vaccination center during the day.
- Those fasting would come to Tao Para Primary School vaccination center before iftar, break fast with the team, and receive vaccination after iftar.
- No other special activity was arranged.

### Questions and Answers

No specific question-and-answer section was recorded in the source for this meeting.

### Source Notes

This was a scheduling meeting rather than a general research-awareness meeting.

## 17. Nobo Muslim Para: Ramadan Vaccination Schedule and Health Camp

| **Date / Time** | **3 February 2025, 11:00 a.m. to 1:30 p.m.** |
| --- | --- |
| Place | Karbari’s house, New Muslim Para, a Tripura-origin Muslim community near Alikadam Sadar and surrounded by Mro communities. |
| Participants / Attendance | 30 participants: 12 men and 18 women. Participants included karbari, villagers, village health worker, group leader, field assistant, mosque imam, and hostel teacher. |
| Purpose | To discuss how vaccination should be conducted during Ramadan. |
| Outcome / Decision | The Ramadan vaccination plan was accepted, and the medical camp increased community satisfaction. |
| Reported by | Chatowai Marma, Group Leader, MVDA, Lama. |

### Agenda / Discussion Points

- Welcome remarks.
- Karbari’s remarks.

### Activities / Proceedings

- Villagers agreed that small children and those not fasting would attend the vaccination center during the day.
- Those fasting would attend Tao Para Primary School vaccination center before iftar, break fast with the team, and receive the vaccine after iftar.
- A medical camp was conducted during the meeting, and villagers received different medicines.

### Questions and Answers

No specific question-and-answer section was recorded in the source for this meeting.

### Source Notes

The source notes villagers were very happy with the medical camp.

## 18. Janali Para: Ramadan Medicine Distribution Schedule and Health Camp

| **Date / Time** | **3 February 2025, 3:30 p.m. to 5:00 p.m.** |
| --- | --- |
| Place | Courtyard of the MVDA village health worker’s house, Janali Para. The para is remote from Alikadam Sadar and inhabited by Muslim community members among Mro and Tripura settlements. |
| Participants / Attendance | 32 participants: 15 men and 17 women. Participants included karbari, MVDA village health worker, group leader, engagement field assistant, mosque imam, and villagers. |
| Purpose | To discuss how the first round of medicine would be given during Ramadan. |
| Outcome / Decision | The community agreed to a Ramadan-compatible medicine distribution arrangement. |
| Reported by | Chatowai Marma, Group Leader, MVDA, Lama. |

### Agenda / Discussion Points

- Welcome remarks.
- Karbari’s remarks.

### Activities / Proceedings

- The group decided that small children and those not fasting would come to the vaccination/medicine center during the day and receive medicine.
- Fasting participants would come before iftar, break fast with the team, and take the medicine after iftar.
- A medical camp was conducted during the meeting, and villagers received medicines.

### Questions and Answers

| **Question / Concern** | **Response / Clarification** |
| --- | --- |
| Will we get malaria medicine free of cost? | Yes. Malaria medicine will be provided free of cost. |
| Who will look after us if there is a problem after taking medicine? | They should contact Shahina Akter, the MVDA worker assigned to the para. She will contact the project team, and necessary action will be taken. |
| Will we get help if we have illnesses other than malaria? | Yes. Support will also be provided for other illnesses as needed. |

### Source Notes

The report states villagers were very happy with the medical camp.

## 19. Krongleng Para: Mixed Community Response to Vaccine and Medicine

| **Date / Time** | **14 January 2025, 6:30 p.m. to 8:10 p.m.** |
| --- | --- |
| Place | Church meeting room, Krangleng Para. |
| Participants / Attendance | 32 participants, including the karbari, five monks/religious leaders, health assistant, Ekota program organizer, engagement coordinator, anthropologist, group leader, field assistant, and villagers. |
| Purpose | To address mixed reactions in the para, where some people wanted the vaccine while others did not want vaccine and medicine. |
| Outcome / Decision | The meeting clarified the purpose of vaccine and medicine, but the source indicates deep-rooted vaccine hesitancy in the community. |
| Reported by | Saleh Ahmed, Engagement Coordinator; Fazle Rabbi, Anthropologist; Chatowai Marma, Engagement Group Leader. |

### Agenda / Discussion Points

- Registration.
- Welcome remarks and introductions.
- Detailed discussion on MVDA research using flipchart.
- Opinion collection.
- Chairperson’s remarks.

### Activities / Proceedings

- The team explained vaccine effectiveness and malaria elimination using flipchart/poster.
- They discussed that many villagers feared vaccine and medicine because they had never taken vaccines and did not vaccinate their children.
- Local health assistant noted that they had been trying to explain vaccination for around 20 years, but villagers still did not vaccinate children.

### Questions and Answers

| **Question / Concern** | **Response / Clarification** |
| --- | --- |
| What will happen if we do not take the malaria vaccine? | Bangladesh aims to eliminate malaria by 2030. Vaccination may help prevent malaria in the area. |
| If malaria happens, we can test and get medicine in the para, so why take vaccine and medicine? | If everyone takes vaccine and medicine, malaria may stop occurring in the para, saving time and money lost due to illness. |
| Government staff ask us to vaccinate our children, but we do not do it. | The health assistant explained that they have been trying for many years, but the community has not vaccinated children. |

### Source Notes

Personal review says the para is about 15 km from Alikadam Sadar and 4 km on foot from Jira Para. Previous meetings showed both community members and the karbari were reluctant. A local religious/community leader helped explain, after which some people agreed to participate.

## 20. Chiuni / Palir Jhiri Para: Ramadan Medicine Intake and Allowance Discussion

| **Date / Time** | **Source is inconsistent: one field says 4 February 2025 at 11:00 a.m.; another says 3 February 2025, 4:00 p.m. to 5:00 p.m.** |
| --- | --- |
| Place | Courtyard of the Ekota para health worker’s house, Chiuni/Palir Jhiri Para. The para is remote from Lama Sadar and inhabited mainly by Muslim community members with Marma communities nearby. |
| Participants / Attendance | 25 participants: 10 men and 15 women. Participants included karbari, villagers, village health worker, group leader, engagement field assistant, and mosque imam. |
| Purpose | To discuss how villagers would take medicine during Ramadan. |
| Outcome / Decision | A Ramadan medicine intake plan was agreed, with assurance that allowances would still be provided. |
| Reported by | Kraingkong Mro, Group Leader, MVDA, Lama. |

### Agenda / Discussion Points

- Welcome remarks.
- Karbari’s remarks.

### Activities / Proceedings

- The meeting focused only on how medicine would be taken during Ramadan.
- The community decided that small children and those not fasting would take medicine during the day.
- Fasting participants would take medicine in the para after iftar.
- No games, drama, or other special activity could be arranged.

### Questions and Answers

| **Question / Concern** | **Response / Clarification** |
| --- | --- |
| If we take medicine in the para, will we still receive the travel allowance that was supposed to be provided? | Yes. Even if medicine is taken in the para, the allowances allocated for participants will be provided. |

### Source Notes

The date and time fields conflict and should be verified before final submission.

# Cross-Cutting Issues Identified Across the Meetings

- Vaccine hesitancy was often linked to a lack of previous vaccination experience, especially in remote paras where EPI or COVID-19 vaccination had not reached many families.
- Fear of signing or giving thumbprints appeared in communities with past mistrust of outside organizations, especially where land-related concerns existed.
- Local-language explanation by karbaris, village health workers, health assistants, and group leaders was repeatedly important for building acceptance.
- Villagers frequently requested assurance about treatment support, transport costs, and follow-up if any side effects occurred.
- Some meetings focused on practical access issues, such as selecting a closer sub-zone or adjusting schedules around Ramadan and fasting.
- Medical/health camps improved acceptance in some communities by providing immediate practical benefit.

# Part 4: Special Meetings During Intervention

Special Meetings During Intervention

Prepared from the original Bangla community engagement records. Para and person names are transliterated from the source where possible.

# Executive Summary

| **Field** | **Details** |
| --- | --- |
| Bakka Para participation report | Vaccine and medicine activity from 19 February 2025 to 24 April 2025. Low first-round participation was linked to rumours, religious/cultural concerns, leadership divisions, and one household moving away. |
| Langchung Para special meeting | Held on 17 February 2025 to address fear and non-participation. Participants were concerned because the para had no prior experience with EPI, COVID-19, or similar vaccination activities. |
| Ponding Para campaign report | Held around the 11 March 2025 camp. Despite repeated information sharing and household invitations, most residents did not participate in the medicine/ongoing service arm. |

# Cross-cutting Issues Identified

- Rumours and misinformation affected participation, including fears about infertility, death, and the “666” mark.
- Communities with little or no prior exposure to vaccination programmes showed higher hesitation and fear.
- Local leadership dynamics influenced turnout, especially where there were multiple karbaris or internal community tensions.
- Repeated interpersonal communication, house-to-house invitation, and involvement of trusted leaders were necessary but not always sufficient.
- Some field teams reported concerns about staff safety and community fatigue when repeated persuasion was attempted.

# Consolidated Meeting Register

| **#** | **Community / Para** | **Date and Time** | **Venue** | **Attendance / Coverage** | **Main Outcome** |
| --- | --- | --- | --- | --- | --- |
| 1 | Bakka Para | Activity start: 19 Feb 2025, 8:30 AM; activity reported through 24 Apr 2025; daily activity time 8:30 AM-4:00 PM. | Lulaing Government Primary School / Lulaing Zone vaccine centre. | 11 households; 72 residents. Round 1: 16 attended, 9 took vaccine/medicine; Round 2: 24; Round 3: 22; unscheduled: 13. | Follow-up engagement increased participation, but first-round turnout was very low due to rumours and leadership/community issues. |
| 2 | Langchung Para, Village ID 002, Tao Para Zone, Lama | 17 Feb 2025, 5:00 PM-6:30 PM. | Yard of the BRAC health worker’s house, Langchung Para. | 18 participants: 5 women and 13 men. | Concerns about vaccine safety and eligibility were discussed; participants agreed to join the research/activity. |
| 3 | Panding Para, Krongleng Para sub-zone | 11 Mar 2025, 9:00 AM-5:30 PM camp. | Camp site beside Krongleng Para. | MVDP census population: 53. Only 5-6 people attended the camp with local representatives. | Most residents declined to participate in the medicine/ongoing service activity despite prior engagement. |

# Detailed Report 1: Bakka Para - Participation in Vaccine and Medicine Activity

| **Field** | **Details** |
| --- | --- |
| Report title | Brief report on participation of Bakka Para residents in the vaccine and medicine activity. |
| Activity date and time | Start date: 19 February 2025 at 8:30 AM. Reported activity period: 19 February 2025 to 24 April 2025. Daily activity time: 8:30 AM to 4:00 PM. |
| Venue | Lulaing Government Primary School / Lulaing Zone vaccine activity centre. |
| Community coverage | According to the census: 11 households and 72 residents. |
| Participation figures | Round 1: 16 people attended the centre, but only 9 took vaccine and medicine. Round 2: 24 people. Round 3: 22 people. Unscheduled: 13 people. |
| Reported by | Lipi Tripura, Engagement Group Leader, Dev Care Foundation, Lama, Bandarban Hill District. |

## Background and Pre-activity Coordination

- Before the vaccine activity started in Lulaing Zone, Engagement Group Leader Mrs. Lipi Tripura arranged a one-day coordination meeting on 14 February 2025 at Lulaing Government Primary School.
- Participants included karbaris from 19 paras, para health workers, BRAC and EktA health workers, engagement field assistants, and medical team members.
- Project Coordinator Dr. Rupam Tripura opened the meeting, thanked participants, and gave overall instructions for the upcoming six-day vaccine and medicine activity.
- The meeting explained the para-wise schedule and requested each karbari and health worker to bring residents to the centre according to the schedule for Round 1, and later for Round 2 and Round 3.

## Purpose

- To contribute to malaria elimination through the “vaccine for all and medicine for all” activity.
- To ensure community participation in the activity.
- To prepare para residents to attend the vaccine centre with their family cards.

## Discussion Topics

11. All residents should participate in the vaccine and medicine activity.
12. The para health worker should visit each household one day before the scheduled date and invite residents to attend the centre.
13. Participants should bring their family cards to the vaccine centre.

## Problems Identified

- Out of 72 potential beneficiaries, only 16 came to the vaccine centre in the first round, and only 9 took vaccine and medicine.
- One of the 11 households had moved away; that household had 7 members.
- Some residents believed that taking the vaccine and medicine could affect women’s ability to conceive.
- Some residents believed that taking the vaccine and medicine would make “666” appear on their forehead.
- Most residents of the para were reported to follow the Christian faith, and religious feelings and traditional beliefs influenced hesitation.
- The para had two karbaris; relatives of the former karbari did not participate, which reduced participation.

## Problem-solving Steps and Outcome

- To improve attendance in Round 2 and Round 3, Medical Assistants Bishwajit Tripura and Ananda Chakma, along with the para karbari, engagement field assistant, and para health worker, visited the para again.
- They held another discussion meeting to explain the activity and address concerns.
- As a result of follow-up communication, some people from the nearby Amtali Para also participated and took vaccine and medicine.
- The activity was implemented jointly by staff from the Upazila Health Complex, BRAC, EktA, and Dev Care Foundation over the three-month activity period.

## Eligibility and Exclusion Criteria Mentioned

- Pregnant women, children under 6 months, and people planning to have a child within one year were not to participate in the activity.
- Other eligible residents could participate in the vaccine and medicine activity.

## Questions and Answers

| **Question / Concern** | **Response / Resolution** |
| --- | --- |
| Will women lose the ability to conceive if they take the vaccine and medicine? | Pregnant women, children under 6 months, and those planning to have a child within one year will not participate. Other eligible residents can participate. |
| Will “666” appear on the forehead after taking vaccine and medicine? | No. This is completely a rumour. |
| Will the next vaccine and medicine activity be held on the same date? | The date will be communicated before the next activity through the engagement field assistant and para health worker. |

# Detailed Report 2: Langchung Para - Special Meeting During Intervention

| **Field** | **Details** |
| --- | --- |
| Community / location | Langchung Para, Village ID 002, Tao Para Zone, Lama. |
| Date and time | 17 February 2025, 5:00 PM to 6:30 PM. |
| Venue | Yard of the BRAC health worker’s house in Langchung Para. |
| Participants | Karbari, Gila Para karbari, member, Assistant Health Inspector, Group Leader, Field Assistant, Para Health Worker, Engagement Coordinator, Anthropologist, religious leader/palak from Gila Para, and residents. |
| Attendance | 18 participants: 5 women and 13 men. |
| Reported by | Saleh Ahmed, Engagement Coordinator; Fazle Rabbi, Anthropologist. |

## Reason for the Meeting

- During vaccine and medicine distribution in the Gila Para sub-zone, residents of Langchung Para did not participate.
- Four residents, including the karbari and the religious leader, came and left without discussing the issue with the team.
- A special meeting was arranged in the para to understand why residents did not want to take the vaccine and medicine and whether they had fear or doubts.
- The broader purpose was to support informed participation, build trust, and improve acceptance of the “vaccine for all and medicine for all” MVDP research activity.

## Main Issues Discussed

- Vaccine effectiveness for malaria elimination.
- Residents’ fear of taking vaccines.
- The fact that the para had reportedly never had EPI, COVID-19, or any other vaccination activity.
- The lack of prior health worker visits for vaccine delivery and the community’s limited understanding of the benefits of vaccination.
- Detailed explanation of the MVDP study using a flipchart/poster, followed by collection of participant opinions and a Q&A session.

## Activities Conducted

- A meeting was conducted with para residents.
- The team discussed the effectiveness and importance of vaccination for malaria elimination.
- The team responded to questions and tried to reduce fear and misunderstanding.
- The meeting concluded with residents agreeing to participate in the research activity.

## Questions and Answers

| **Question / Concern** | **Response / Resolution** |
| --- | --- |
| We have never taken vaccines before. Will it be painful? | The the vaccine had undergone safety assessment and has no major side effects. Like other vaccines, mild redness, swelling, pain at the injection site, mild fever, drowsiness, or convulsion-like symptoms may occur, but these are commonly associated with vaccines. |
| Does everyone have to take the vaccine? | Yes. Residents of the para who give informed consent and are over 6 months of age will receive the vaccine. |
| People of Gila Para are receiving only vaccine. Why will we receive both vaccine and medicine? Please give us vaccine as well. | The study aims to assess the effectiveness of both vaccine and medicine. Taking both will not harm a person. Vaccine and medicine will not be provided only in this para; 25 paras across Lama and Alikadam will receive vaccine and medicine. The team will remain available for any health service needs. |

## Outcome

- The importance of vaccination for malaria elimination was explained.
- Residents agreed during the meeting to participate in the research activity.

# Detailed Report 3: Ponding Para - Medicine and Ongoing Service Camp Response

| **Field** | **Details** |
| --- | --- |
| Community / location | Ponding Para, Krongleng Para sub-zone. |
| Date and time | 11 March 2025, camp conducted from 9:00 AM to 5:30 PM. |
| Venue | Camp site near Krongleng Para. |
| Programme arm | “Medicine for all and ongoing service” arm of the MVDP programme. |
| Population / coverage | According to the MVDP census, 53 residents live in Panding Para. |
| Participants present at camp | Only 5-6 residents attended with the karbari, MVDP Village Health Worker, and BRAC health worker. |
| Reported by | Chathowai Marma (Hiro), Engagement Group Leader, MVDP, Dev Care Foundation. |

## Purpose

- Residents had previously indicated they would participate in the research, but later said they would not take the medicine.
- The camp aimed to support informed participation of all relevant residents in the “medicine for all and ongoing service” activity.
- The objective was for Ponding Para residents to attend the camp and receive medicine and other services.

## Steps Taken Before and During the Camp

14. The para karbari, MVDP Village Health Worker, and BRAC health worker were informed about the “medicine for all and ongoing service” programme several times through meetings and materials.
15. Before the camp, an engagement field assistant informed the para that medicine and other services would be provided at Krongleng Para on 11 March 2025.
16. An evening meeting was held with all para residents to remind them about the upcoming camp and services.
17. After the evening meeting, the MVDP Village Health Worker went house to house at night and invited residents to go to the Krongleng Para sub-zone camp.
18. Although residents were expected to gather and attend the camp in the morning, most people went to their own work and those who remained in the village also did not want to come.

## Problems Observed at the Camp

- Only 5-6 people attended the camp with the MVDP Village Health Worker, karbari, and BRAC health worker.
- Even among those who came, difficulties were observed during medicine intake: some did not want to swallow the medicine, some held it in the mouth, and some spat it out.
- After waiting for additional participants, the team asked the local workers and karbari why no one else was coming. They stated that the village people would not come and that they did not want to participate in MVDP activities.
- When asked whether another village-level meeting or village-based medicine intake would help, the local representatives said residents were annoyed and that visiting the village in the current situation could create risk or accidents. They also said they could not take responsibility if anything happened.

## Discussion Questions and Responses

| **Question / Concern** | **Response / Resolution** |
| --- | --- |
| Why did the para residents not come? | According to local representatives, the villagers had no interest in MVDP activities and were not interested in taking medicine. |
| Do the residents still have a gap in understanding? | The response was that the engagement field assistant and others had explained many times; they believed there was no major remaining gap in information or understanding. |
| What kind of incident could occur if the team visited the village? | Local representatives warned that villagers might attack the team because they had become irritated by repeated explanations. |
| What is the opinion of the community and local representatives? | The villagers’ position was that they would not participate in MVDP activities. The local representatives said they would follow whatever decision Dev Care Foundation makes. |

## Personal Review / Observations

- Ponding Para is mostly made up of the karbari’s own relatives; most residents are related to each other.
- The source notes that the karbari and some MVDP workers themselves showed little interest, although they had previously expressed interest in receiving benefits.
- Residents reportedly believed rumours that taking medicine could cause different problems, death, or reduced ability to conceive.

## Outcome

- Despite repeated engagement, meeting, household invitation, and camp preparation, most residents did not participate.
- The report recommends that the final decision be made by Dev Care Foundation, as local representatives stated that they would accept the organization’s decision.

# Overall Recommendations from the Structured Review

- Use locally trusted leaders, health workers, and religious/community influencers early in the process before intervention day.
- Prepare rumour-specific messages on infertility, death, religious marks, and vaccine side effects, and repeat them in simple local language.
- Document leadership disputes or internal village politics before campaign scheduling, as they can affect attendance.
- For low-exposure communities, hold separate vaccine-literacy sessions before asking for participation.
- Where field safety concerns are raised, avoid unscheduled visits and escalate to programme leadership for a risk-managed approach.

# Part 5: Special Meetings Before Booster Vaccination

Special Meetings Before Booster Vaccination

Project context: Malaria elimination research / MVDA community engagement records

# Executive Summary

- The file contains six community-level special meeting records held before the planned booster vaccination round.
- The main purpose was to re-inform para residents about the booster dose, collect community feedback after earlier vaccine/medicine rounds, reduce fear or misconceptions, and support informed participation at the vaccination centers.
- Most communities reported that they would participate in the booster round after discussion. Several communities also reported fewer malaria cases after the earlier vaccination activities.
- Common questions focused on booster eligibility, whether one-dose recipients can receive the booster, whether people who missed previous doses can join, timing and location of the booster round, side effects, and whether current-service paras would receive vaccination.
- Logistical concerns were noted in remote paras, especially where the vaccination center was far away or difficult to reach with children.

# Meeting Register / Summary Table

| **No.** | **Para / Area** | **Date** | **Time** | **Attendance** | **Venue** | **Main Focus** |
| --- | --- | --- | --- | --- | --- | --- |
| 1 | Ramju Para | 02 Dec 2025 | 7:00 AM-11:00 AM (source header also shows 11:00 AM) | 21 (8 male, 13 female) | Large banyan tree in the para | Re-orientation before booster dose; feedback after vaccine and medicine round. |
| 2 | Gila Para | 02 Dec 2025 | 2:30 PM-4:15 PM | 31 (20 male, 11 female) | Yard of the pastor/religious leader’s house | Community agreed to attend booster round; malaria cases reportedly reduced. |
| 3 | Hamuk Para / Lalu Karbari Para area | 08 Dec 2025 | 3:30 PM-7:30 PM | 45 (40 male, 5 female) | Karbari’s yard under a tamarind tree | Low earlier participation addressed; fear reduced after earlier doses. |
| 4 | Kai Ang Para | 08 Dec 2025 | 7:30 PM-8:30 PM | 40 (15 male, 25 female) | Yard of a BRAC worker’s house | Discussed closer booster point due to long travel to Krangleng Para center. |
| 5 | Mensing Para | 15 Dec 2025 | 7:00 PM-8:30 PM | 26 (20 male, 6 female) | Central Chhera/community house | Discussed booster participation despite family dispute between karbari and village health worker. |
| 6 | Thandajhiri Para | 08 Dec 2025 | 6:30 PM-8:00 PM | 37 (20 male, 17 female) | Karbari’s house | Follow-up due to low attendance in first, second, and third dose rounds. |

# Cross-cutting Issues Identified

- Booster eligibility was the most frequent concern. The recorded response was that people who received at least one previous malaria vaccine dose would be eligible for the booster dose.
- New residents or people who never received any previous malaria vaccine dose would not receive the booster dose, but they would continue to receive other available health services.
- Several communities asked whether mild side effects such as fever, body ache, or appetite changes were expected. The response recorded was that mild fever and body pain may occur and usually resolve within two to three days.
- Remote paras requested more accessible vaccination points. Kai Ang Para specifically reported that the previous center was about two hours away on foot and difficult for families with small children.
- Community leaders and residents in several paras reported that malaria cases had reduced compared with the previous year after earlier vaccination activities.
- Medical camps, light refreshments, first-aid/primary medicine distribution, or medical support were conducted in several meetings; no sports, drama, or special events were organized.

# Common Recommendations

19. Notify each para again before the booster date through field assistants and village health workers.
20. Use karbaris, village health workers, BRAC/Ekta workers, and respected elders to explain booster eligibility and reduce confusion.
21. For remote paras, select the closest feasible vaccination point and communicate the route clearly before the booster day.
22. Continue medical camp support during meetings where possible, especially in paras that asked for treatment support.
23. Clarify that the booster round is for people who previously received at least one malaria vaccine dose, while other health services remain available for the wider community.
24. Record unresolved data issues separately, especially inconsistent dates and uncertain transliteration of para names.

# Detailed Meeting Reports

## 1. Ramju Para - Booster Dose Orientation

| **Date and Time** | **02 December 2025. The source states 7:00 AM to 11:00 AM; the header also lists 11:00 AM.** |
| --- | --- |
| Attendance | Total 21 participants: 8 male and 13 female. Participants included karbari, para residents, MVDA village health worker, group leader, and engagement field assistant. |
| Venue | Under a large banyan tree in Ramju Para. The place is commonly used by residents for rest, social discussion, and religious/social activities. |
| Purpose | To inform all residents again about the upcoming booster dose, collect opinions after vaccine and medicine activities, and support informed participation in the booster vaccination round. |
| Group / Para Type | Ramju Para was recorded as a vaccine and medicine arm para. |

### Agenda / Discussion Topics

- Introduction and welcome remarks.
- Collection of residents’ feedback after receiving vaccine and medicine.
- Discussion on booster dose.
- Karbari’s closing remarks and formal meeting closure.

### Meeting Activities and Main Discussion

The field assistant requested a meeting before the booster dose so that all eligible residents would come to the vaccination center.

A medical camp was conducted in the para during the meeting.

### Questions and Answers

| **Question** | **Answer / Response** |
| --- | --- |
| If someone received only one vaccine dose, will that person receive the booster dose? | Yes. The recorded response was that people in the para who received even one vaccine dose would receive the booster dose. |

### Outcome / Follow-up

- The community was reminded that the booster dose may start from January as recorded in the source narrative.
- Participants were encouraged to attend the vaccination center for the booster round.
- The source does not record additional questions beyond the one-dose eligibility question.

Reported by: Kraingkrong Mro, Group Leader, MVDA, Lama. Report date: 02 December 2025.

## 2. Gila Para - Booster Dose Orientation and Feedback Meeting

| **Date and Time** | **02 December 2025, 2:30 PM to 4:15 PM.** |
| --- | --- |
| Attendance | Total 31 participants: 20 male and 11 female. Participants included karbari, para residents, MVDA village health worker, group leader, and engagement field assistant. |
| Venue | Yard of the pastor/religious leader’s house. A tarpaulin was spread for seating. The source notes that this venue is commonly used for social and religious activities. |
| Purpose | To remove doubts and misconceptions before the booster dose, support informed participation, and collect feedback after previous vaccination. |
| Community context | Gila Para was recorded as a vaccine arm para with an estimated population of 316. Most residents had previously received vaccine doses. |

### Agenda / Discussion Topics

- Introduction and welcome remarks.
- Collection of feedback after vaccination.
- Discussion on the booster dose.
- Karbari’s remarks and meeting closure.

### Meeting Activities and Main Discussion

The field assistant requested the meeting before the booster dose so that all residents would come to the vaccination center.

Residents and the karbari stated that they would receive the booster dose.

Community members stated that the number of malaria cases in the para had decreased after vaccination.

A medical camp was conducted in the para during the meeting.

No drama, games, or similar activity was organized.

### Questions and Answers

| **Question** | **Answer / Response** |
| --- | --- |
| If someone received only one vaccine dose, will that person receive the booster dose? | Yes. People who received even one vaccine dose in the para would receive the booster dose. |
| When will the vaccine be given? | The field assistant will visit the para before vaccination and inform everyone. |

### Outcome / Follow-up

- Residents expressed willingness to participate in the booster dose round.
- The meeting reinforced that vaccination timing would be communicated before the campaign.

Reported by: Moni Mukul Chakma, Group Leader, MVDA, Lama. Report date: 02 December 2025.

## 3. Hamuk Para / Lalu Karbari Para Area - Follow-up Meeting for Low Prior Participation

| **Date and Time** | **Source heading lists 08 December 2025 at 2:30 PM; the time row records 3:30 PM to 7:30 PM and also mentions 02 December 2025. This has been kept as a source inconsistency.** |
| --- | --- |
| Attendance | Total 45 participants: 40 male and 5 female. Participants included karbari, para residents, MVDA village health worker, group leader, and engagement field assistant. |
| Venue | Karbari’s yard under a tamarind tree. The karbari advised holding the meeting there because the place is used for social and religious activities. |
| Purpose | To encourage booster dose participation because Hamuk Para had comparatively low participation in earlier vaccine rounds. |
| Community context | Hamuk Para and Lalu Karbari Para were described as adjacent paras. Hamuk Para was recorded as a vaccine group para. |

### Agenda / Discussion Topics

- Introduction and welcome remarks.
- Collection of feedback after vaccination.
- Discussion on booster dose.
- Karbari’s statement and meeting closure.

### Meeting Activities and Main Discussion

The source states that previous vaccine participation in Hamuk Para was lower than in other paras, so the booster dose was discussed again.

Residents stated that they had no serious problems after vaccination. Some had mild fever or body ache.

Some residents had been afraid before the vaccine, but the fear had reduced after prior experience.

Residents and the karbari said everyone would receive the booster dose.

A medical camp was conducted during the meeting; no drama, games, or other special activity was organized.

### Questions and Answers

| **Question** | **Answer / Response** |
| --- | --- |
| Will people who did not receive any vaccine dose before receive the vaccine now? | No. People who did not receive any previous vaccine dose will not receive the booster dose. |
| Will current-service paras receive vaccine? | Not at this stage. The para will be informed before any vaccination is provided later. |
| Malaria has decreased after the malaria vaccine, but other diseases have not decreased. Why? | The vaccine mainly helps protect against malaria infection; it does not work for other diseases. If residents have other illnesses, Dev Care will help them receive treatment. |

### Outcome / Follow-up

- Fear about the vaccine appeared to reduce after earlier rounds.
- The community said they would participate in the booster round.
- The main eligibility rule was clarified: the booster is for those who had received at least one earlier malaria vaccine dose.

Reported by: Kraings Mro, Group Leader, MVDA, Lama. Report date: 08 December 2025.

### Source Notes

- The source contains an internal date inconsistency for this entry: 08.12.2025 in the heading and 02.12.2025 in the time row.

## 4. Kai Ang Para - Accessibility Discussion Before Booster Dose

| **Date and Time** | **Source heading lists 08 December 2025 at 2:30 PM; the time row records 7:30 PM to 8:30 PM and also mentions 02 December 2025. This has been retained as a source inconsistency.** |
| --- | --- |
| Attendance | Total 40 participants: 15 male and 25 female. Participants included karbari, para residents, MVDA village health worker, group leader, and engagement field assistant. |
| Venue | Yard of a BRAC worker’s house in Kai Ang Para. The source records that the para has 12 families and the BRAC worker’s house was large enough for a night meeting and medical camp. |
| Purpose | To re-inform residents about the booster dose and understand whether the previous vaccination center created access problems. |
| Community context | Residents had to walk about two hours from Kai Ang Para to Krangleng Para vaccination center for the first, second, and third rounds. This was difficult for families with small children. |

### Agenda / Discussion Topics

- Introduction and welcome remarks.
- Feedback after previous vaccination.
- Discussion on booster dose and suitable vaccination location.
- Karbari’s statement and meeting closure.

### Meeting Activities and Main Discussion

Residents asked whether the booster could be given inside their own para. The team explained that carrying all materials by foot into the para would be difficult.

After discussion, residents agreed to come to a nearby para recorded as Re Ang Para / Ri Ang Para in the source. The transliteration is uncertain due to source encoding.

Residents said they had no major problems after vaccination. Some reported mild fever or body ache; some could not work in the jhum for three to four days.

Fear about vaccination had reduced, and residents said they would take the booster dose.

A medical camp was conducted during the meeting. No drama, games, or special activity was organized.

### Questions and Answers

| **Question** | **Answer / Response** |
| --- | --- |
| Why do people lose appetite after vaccination? | The team stated that they had no evidence that vaccination causes loss of appetite. Mild fever or body ache may occur and usually improves within two to three days. |
| Will vaccine and medicine both be given this time? | Only the booster dose will be given this time. Medicine will not be administered. |
| People who did not receive vaccine earlier but are now over six months old - will they receive vaccine? | No. People who did not receive any previous vaccine dose will not receive the booster. People who received at least one prior vaccine dose will receive the booster. |

### Outcome / Follow-up

- The community accepted the need for a nearer vaccination point rather than the earlier distant center.
- The main eligibility rule for booster dose was clarified.
- Community members said they would participate in the booster round.

Reported by: Chathowai Marma, Group Leader, Kurukpata Zone, MVDA, Lama. Report date: 08 December 2025.

## 5. Mensing Para - Booster Preparation and Community Relationship Follow-up

| **Date and Time** | **15 December 2025, 7:00 PM to 8:30 PM.** |
| --- | --- |
| Attendance | Total 26 participants: 20 male and 6 female. Participants included the Mensing Para karbari, residents, village health worker, group leader, and field assistant. |
| Venue | Central Chhera / community house in Mensing Para. The source records 25 families and states that para-level meetings are generally held there. |
| Purpose | To prepare for the booster dose and address possible issues caused by a family dispute between the karbari and the MVDA village health worker. |
| Community context | The meeting also discussed current malaria status and treatment issues in the para. |

### Agenda / Discussion Topics

- Greeting and introduction.
- Discussion on malaria situation and para-level treatment issues.
- Discussion on booster dose.
- Open discussion and feedback collection.
- Thanks and closure.

### Meeting Activities and Main Discussion

The facilitator introduced Dev Care Foundation and summarized its work.

The BRAC health worker reported that malaria cases had decreased compared with earlier periods, especially PF malaria.

The meeting discussed how the first, second, and third doses work and how the booster dose would help.

After discussion, participants stated that they would receive the booster dose.

Those who had received one dose stated they would come early to the center, as in the previous round.

Light refreshments and primary medicine were distributed after the meeting; no drama or games were organized.

### Questions and Answers

| **Question** | **Answer / Response** |
| --- | --- |
| Will people who received only one dose also receive the booster dose? | Yes. Everyone who received at least one malaria vaccine dose under the MVDA program can receive the booster dose. |
| Will people who came to this para from another para receive vaccine? | Newly joining residents cannot be added to the MVDA booster activity here. If the para they came from has the MVDA activity, they may receive services there. |
| Will people who never received a vaccine dose receive treatment? | Yes. People living in the para who could not receive vaccine for other reasons can receive medical services from the office. |
| Will people who were previously pregnant or breastfeeding receive vaccine now? | No. The source records that they will not receive the vaccine, but they will receive other services. |

### Outcome / Follow-up

- Community members assured participation in the booster dose round.
- The meeting addressed concerns that family disputes might affect booster participation.
- Other health service access was clarified for people not eligible for booster vaccination.

Reported by: Chathowai Marma (Hiro), Engagement Group Leader, MVDA, Kurukpata Zone, Alikadam. Report date: 16 December 2025.

## 6. Thandajhiri Para - Follow-up for Low Attendance in Earlier Rounds

| **Date and Time** | **08 December 2025, 6:30 PM to 8:00 PM.** |
| --- | --- |
| Attendance | Total 37 participants: 20 male and 17 female. Participants included karbari, para residents, MVDA village health worker, group leader, and engagement field assistant. |
| Venue | House of the karbari in Thandajhiri Para. The source records that the para has 15 families and para-level meetings are usually held at the karbari’s house. |
| Purpose | To increase participation in the booster dose round because attendance in the first, second, and third malaria vaccine doses had been low. |
| Eligibility focus | The meeting emphasized that people who received at least one previous dose should participate in the booster dose. |

### Agenda / Discussion Topics

- Greeting and introduction.
- Identification of current malaria and other disease problems in the para.
- Discussion on booster dose.
- Open discussion and feedback collection.
- Thanks and closure.

### Meeting Activities and Main Discussion

Participants and BRAC staff agreed that malaria cases had decreased compared with the previous year.

Residents thanked the project and related organizations for support with scabies and other treatment services in the para.

The booster dose and the dose-wise function of the vaccine were explained in detail.

Residents who still had doubts after receiving the first dose said they would receive the booster dose after discussion.

The karbari requested another meeting one day before the booster dose.

Light refreshments and a medical camp were provided after the meeting; no drama, games, or similar activity was organized.

### Questions and Answers

| **Question** | **Answer / Response** |
| --- | --- |
| Will new people receive this vaccine? | No. Only people who received at least one malaria vaccine dose will receive the booster dose. New people will not receive the booster, but all other services will be available to new and old residents. |
| Why are medicines not provided to the para-level worker? | The team stated that medicine distribution to para-level workers has not stopped, but medicines are not supplied in large quantities as before. If the village has high medicine need, the team will try to provide support. |
| Will the office provide mosquito nets? | The team works on malaria but does not directly distribute mosquito nets. If arrangements can be made through BRAC or Ekta via the office, nets may be provided. |

### Outcome / Follow-up

- Booster eligibility and non-eligibility for new people were clarified.
- Participants requested additional advance communication before the booster round.
- Demand for mosquito nets and medicine support was recorded for follow-up.

Reported by: Ushanta Chakma, Medical Assistant, MVDA. Report date: 08 December 2025.

Dev Care Foundation

Reg. No. S-2863(376)/2002

1501/B, O. R. Nizam Road, Mehedibag, Chattogram, Bangladesh

Rumor Identification and Cause Investigation Report

# 1. Visit and Initial Observation

On 24 January 2025, before the start of vaccine and medicine distribution activities in Lulaing Zone, Dr. Rupam Tripura, Project Coordinator; Dr. Ibrahim Khalil, Research Physician; Himadri Chakma, Lab In-Charge; Moni Mukul Chakma, Group Leader; and I, Saleh Ahmed, Engagement Coordinator, visited the Lemupalang sub-zone and Lulaing Government Primary School.

Alongside the zone visit, we communicated personally with the MVDA para health workers, engagement field assistants, and community residents. During the visit to Lemupalang sub-zone, an MVDA para health worker, Naing Mro, was with us. He works in the New Dewan Para vaccine group.

We asked him about the situation in his para and the level of interest among residents in receiving the vaccine. In response, the para health worker mentioned several problems. One of the main issues was related to a local person named Kaimpre Mro.

# 2. Person Reported as Spreading the Rumor

| **Name** | **Kaimpre Mro** |
| --- | --- |
| Age | Approximately 30 years |
| Education | HSC passed |
| Occupation | Timber/wood businessman |
| Local involvement | Involved in local politics |
| Residence | Kewaju Para, a nearby market-area para |
| Connection with study areas | He travels to different paras for business and has regular contact with Dewan Para, which is included in the vaccine group of the study. |

The above-mentioned person has direct contact with residents of different paras and is familiar with the villages included in the research. He reportedly circulated misleading information about the project among the villagers.

## Reported rumor statement

According to the report, approximately 15 to 20 days earlier, he told villagers words to the following effect:

"Since this vaccine is being used for research, and you do not know how to read and write, you may face various problems later if you take this vaccine. They will vaccinate you and leave, and afterward you will not be able to find them."

# 3. Effect of the Rumor in Dewan Para

Because of this person, hesitation and disagreement have developed among the residents of Dewan Para regarding vaccine acceptance. A difference of opinion has appeared between the villagers and the karbari. For example, even though the karbari wants to receive the vaccine, the villagers have taken a position against receiving it.

# 4. Direct Discussion with Kaimpre Mro

To address the situation and observe the matter more deeply, we contacted Kaimpre Mro directly. We sat with him at Kewaju Para Bazar and tried to understand his personal viewpoint regarding this research. During the discussion, he shared several personal opinions about the research activities:

25. Since this vaccine is for research, why is it being provided only to people of the Mro community? He also asked why it is not being provided in Rangamati and Khagrachhari districts.
26. If any problem occurs among the villagers five or six years after vaccination, the project team may no longer be present. In that case, who will the villagers contact?
27. He said that he needs to think more and search on Google to learn more before supporting vaccination. He stated that if he tells the villagers to take the vaccine and they later face any problem, he will not be able to face them. He said that he does not want anyone to be harmed because of him.

# 5. Clarification Provided by the Project Team

The team members held a broader discussion with him. Dr. Ibrahim Khalil, Research Physician, explained the MVDA project activities in detail. The following points were discussed:

- Among the three hill districts, Bandarban has the highest malaria infection rate.
- Among the 100 paras under this research, there are 7 Bangali paras, 75 Mro paras, 16 Tripura paras, 1 Marma para, and 1 Chakma para.
- No bias or preference toward any particular community was applied in the selection of paras/villages.
- The paras were selected through lottery, considering the malaria infection rate.
- The vaccine has been proven to be safe. There are no significant side effects reported so far.
- After vaccination, some mild symptoms may occur, such as light fever, loss of appetite, irritability, or drowsiness.
- There may also be redness, swelling, or pain at the injection site.
- Up to the time of reporting, no major side effects had been reported after vaccination.

# 6. Outcome of the Discussion

After the discussion between both sides, Kaimpre Mro said that he does not want any obstruction to occur in the project activities because of him and that he will not cause any harm. In this context, we requested his overall cooperation in project implementation.

We also provided him with a mobile number so that he can contact us at any time if he has questions or wants to know anything further. He was invited to visit our Lama office, and he expressed interest in coming to Lama.

# 7. Follow-up Health Camp and Meeting

On 26 January 2025, a health camp was conducted at the Lemupalang sub-zone community clinic by Dr. Imtiaz, Research Physician, and three Medical Assistants. A total of 75 patients received medical treatment there.

At the same time, a meeting was held with six karbaris, a headman, a member, villagers who came for treatment from the seven paras included under that sub-zone, and Kaimpre Mro, the person reported to have spread the rumor. The MVDA study was discussed in detail during the meeting.

The headman, karbaris, member, and villagers expressed willingness to participate in the research. However, according to the report, Kaimpre Mro was attempting to create a problem by telling various misleading statements to the villagers.

# 8. Request for Action and Advice

Considering the above situation, the reporter requested necessary action and appropriate advice to address the rumor so that the issue could be resolved before starting the vaccine and medicine distribution program.

# 9. Reporter

| **Reported by** | **Saleh Ahmed** |
| --- | --- |
| Designation | Engagement Coordinator |
| Organization | Dev Care Foundation |
| Date | 27 January 2025 |

# Suggested Follow-up Actions Based on the Report

- Hold a targeted follow-up meeting in Dewan Para with the karbari, headman/member, para health worker, engagement staff, and trusted local representatives.
- Address the exact rumor messages directly and clearly, especially concerns about research, long-term safety, and future contact points.
- Share project contact numbers and explain the follow-up support mechanism to residents before vaccination begins.
- Invite Kaimpre Mro to the Lama office or another formal briefing so that his questions are answered transparently and he can avoid spreading further confusion.
- Use the health camp platform to reinforce accurate information because villagers already gathered there for medical services.
